# Assessment and management of midfoot osteoarthritis by podiatrists in Australia: a cross-sectional survey of current practice

**DOI:** 10.1007/s00296-025-05881-9

**Published:** 2025-05-12

**Authors:** Polly Q. X. Lim, Hylton B. Menz, Karl B. Landorf, Michelle R. Kaminski, Kade L. Paterson, Shannon E. Munteanu

**Affiliations:** 1 Discipline of Podiatry, School of Allied Health, Human Services and Sport, La Trobe University, Melbourne, VIC 3086 Australia; 2 Department of Podiatry, Monash Health, Melbourne, VIC 3168 Australia; 3 School of Primary and Allied Health Care, Monash University, Melbourne, VIC 3199 Australia; 4 Department of Physiotherapy, Centre for Health, Exercise and Sports Medicine, School of Health Sciences, The University of Melbourne, Melbourne, VIC 3010 Australia

**Keywords:** Midfoot, Osteoarthritis, Podiatry, Assessment, Management, Surveys and questionnaires

## Abstract

Midfoot osteoarthritis (OA) is a common and disabling condition, yet lacks clinical guidelines for its assessment and management. This study aimed to investigate strategies used by Australian podiatrists to assess and manage midfoot OA. An exploratory cross-sectional survey of podiatrists working in Australia was conducted. Respondents were invited through professional organisations to complete an online questionnaire on their usual assessment and management practices for midfoot OA, based on a vignette. Between November 2022 and July 2023, 103 participants (52% women, mean age 40 years) with an average of 15 years of clinical experience responded. Physical assessments (including history taking and musculoskeletal assessments) (96%) and medical imaging (90%) were commonly used diagnostic methods, with palpation (96%), passive movement testing (95%) and x-ray imaging (62%) frequently reported. Associated impairments were assessed with range of motion testing of the midfoot (96%), gait analysis (95%), functional tests (83%), muscle strength (72%), and observation of static alignment (71%), including foot posture (69%). Most participants assessed comorbidities (93%), body composition (66%) and cognitive/psychosocial impairments (62%). Treatment approaches included footwear advice (97%), orthotic therapy (85%), strapping/taping (81%), education (79%), and exercise therapy (75%). One-third (34%) expected a ‘satisfactory’ treatment response within two months, and 36% would refer to another healthcare practitioner for co-management between two and three months if treatment was ineffective. A wide range of approaches are used to assess and manage midfoot OA. These findings have potential to inform the design of future studies in developing valid assessment approaches and effective interventions for this condition.

**Supplementary Information:**

The online version contains supplementary material available at 10.1007/s00296-025-05881-9.

## Introduction

Foot osteoarthritis (OA) is a significant cause of pain and disability in older people [1]. Midfoot OA is one of the most common forms of OA, affecting 1 in 8 adults aged 50 years and over [1]. People with midfoot OA report impaired physical [2–4] and mental [2, 4] wellbeing, with more than 80% reporting the condition to be disabling [4]. The aetiology of midfoot OA is not well understood but is likely to be multifactorial, and is associated with increased age, female sex, being in routine/intermediate occupations, obesity, pain in other weightbearing joints, the presence of non-musculoskeletal comorbidities, as well as previous foot and ankle injury [4].

Despite the prevalence and impact of midfoot OA, the evidence guiding its assessment and management is limited. There is no universally agreed definition for the clinical diagnosis of midfoot OA [5–7]. Symptoms have been described as dorsal [6, 8, 9], plantar [6], or medial midfoot pain [2, 8]; exacerbated by weightbearing activities [9], including walking on uneven surfaces, stairs [10–12] or barefoot [2]; and characterised by stiffness in the morning lasting less than 30 min [13, 14]. The condition also often involves multiple midfoot joints, which can lead to ambiguous clinical signs [6, 10, 11]. Diagnostic methods such as inspection and palpation for dorsal bony prominences and passive movement testing have been used [7, 10, 11, 13, 14], but the accuracy of these approaches is uncertain [7]. In addition, people with midfoot OA may display reduced muscle strength [3], altered foot structure and function [15–17] and limited foot joint mobility [6, 7], so assessments for these deficits have been recommended to support treatment planning [3, 6, 15, 17]. Radiological assessment, particularly plain film x-ray imaging, is recommended to confirm diagnosis when there is uncertainty [6, 10, 11, 16, 18, 19]. However, other radiological approaches including magnetic resonance imaging (MRI) [14, 20, 21], musculoskeletal ultrasound [20–24], weightbearing cone-beam computed tomography [21, 25], single-photon emission computed tomography [10, 11, 26], and technetium-99 m bone scans [10, 27] have also been used to aid diagnosis. Before designing research studies or guidelines for midfoot OA, an important initial step is to determine how midfoot OA is typically assessed in clinical practice and the impairments that may commonly present with the condition.

Establishing best care for midfoot OA is challenging due to limited evidence. Several non-surgical interventions including general analgesic advice [28–30], shoe stiffening inserts [9, 31], arch contouring foot orthoses [8, 32], intra-articular corticosteroid injection [22, 33], and appropriate footwear [34] have been recommended; however, the lack of randomised controlled trials evaluating interventions for this condition remains a limitation [35]. Appropriately powered clinical trials are essential to evaluate the efficacy of these interventions. However, foundational research is required to define usual care for midfoot OA [5]. This knowledge would facilitate decisions to select what interventions to evaluate in future trials. Therefore, to ensure that future clinical trials are broadly applicable, the next logical step is to identify the interventions employed by clinicians who commonly manage foot conditions non-surgically. In the United Kingdom, most people with midfoot OA initially seek care from general practitioners and podiatrists for their foot-related concerns [4]. In Australia, general practitioners commonly use pharmacological approaches to manage foot and ankle OA [29]. However, there is currently no evidence regarding the assessment and management of midfoot OA by podiatrists. Therefore, the aim of this study was to explore the current approaches of podiatrists in Australia for assessing and managing midfoot OA. This study represents an important initial step in identifying usual practices of podiatrists who assess and manage people with midfoot OA, providing valuable guidance for clinicians and researchers. It is envisaged that the findings will guide clinical decisions and future research for the assessment and management of midfoot OA.

## Methods

### Design

This study was a cross-sectional online survey of Australian-based podiatrists’ current clinical practice for assessing and managing midfoot OA and is reported in accordance with the Consensus-Based Checklist for Reporting of Survey Studies (CROSS) [36] and the recent guideline by Zimba and Gasparyan [37]. Ethical approval was obtained from the La Trobe University Human Research Ethics Committee (reference number: HEC22329).

### Participants

Between November 2022 and July 2023, the link to the REDCap™ (Research Electronic Data Capture, Vanderbilt University) [38, 39] survey was advertised through the Australian Podiatry Association, and partner organisations (e.g., hospital podiatry departments, local podiatry clinics, and university podiatry programs). We also advertised the survey link on social media platforms (i.e., Facebook, ‘X’ formally ‘Twitter’ and LinkedIn). To qualify for the study, participants had to be registered podiatrists practicing in Australia and have treated at least one patient with midfoot OA in the previous six months. A sample size calculation, based on n = 5952 currently registered podiatrists in Australia (as of September 2022) and a 95% confidence interval and 10% margin of error, resulted in an estimated minimum sample size of 95 participants to obtain a representative sample of the profession [40, 41]. Therefore, we aimed to collect responses from a minimum of 100 podiatrists [42], with representation distributed proportionately across Australia relative to the number of registered podiatrists in each state or territory [41]. The sample size for this study is consistent with a previous survey of podiatrists and physical therapists regarding their management of first metatarsophalangeal joint OA [42].

### Procedures

**Fig. 1:**
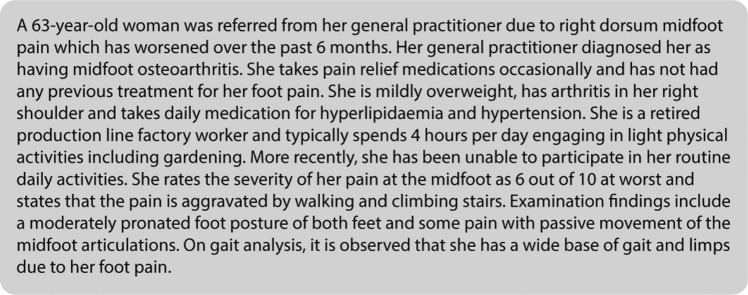
Case vignette of a typical patient with symptomatic midfoot OA

The survey was shared with participants via an online link created in REDCap™ [38, 39] that included the purpose of the study, the informed consent statement, and the survey questions. The survey questions were formulated to explore the participants’ methods for diagnosing, assessing, and managing midfoot OA. Prior to these questions, a case vignette portraying a typical patient with midfoot OA was presented to provide context (Fig. 1). A case vignette is a common and valid tool used for assessing clinical practices [43, 44]. We followed a similar approach to a survey conducted for first metatarsophalangeal joint OA [42]. In the development phase of the survey, we conducted pre-testing of the survey by asking four experienced podiatrists (clinical experience ranging from 10 to 24 years) who frequently treat people with musculoskeletal conditions to provide feedback on the flow, clarity, objectivity and content of the survey. The survey was reported to be feasible, relevant and complete. Suggestions to improve the user interface and grammatical errors were addressed. Three rounds of meetings attended by PQXL, SEM, KBL, HBM and KLP were conducted to finalise the survey and the average time to complete the survey during the pilot testing was 20 min.

The survey was structured into five main sections (Supplementary file 1). The first section consisted of general participant characteristic questions regarding gender, age, qualification(s), and year(s) qualifications obtained; work setting(s), years of clinical experience, primary role, postgraduate training, and endorsement status for prescribing scheduled medicines. The remaining sections of the survey began with a midfoot OA case vignette as mentioned above. Sections two and three focused on diagnostic and assessment approaches for identifying associated factors, physical impairments, and comorbidities. The fourth section explored participants’ typical treatment approaches for managing midfoot OA, including interventions, referrals, and considerations for weight loss. The fifth section evaluated participants’ expectations regarding the timeline for managing the patient described in the case vignette, focusing on treatment outcomes and anticipated progress over time. This section explored their treatment expectations on a typical timeline for managing the patient in the case vignette. For each section, participants had the opportunity to select from a pre-defined list of options and provide suggestions for additional diagnostic tools, assessments, or treatment approaches that were not covered in the survey options.

Survey responses were collected and managed using the REDCap™ electronic data capture tool hosted at La Trobe University (Australia) [38, 39]. REDCap™ is a secure, web-based software platform that provides an intuitive interface for validated data capture, audit trails for tracking data manipulation to preventing unauthorised access; supports automated export procedures to common statistical packages, data integration and interoperability with external sources [38, 39]. As the survey responses were kept anonymous, we manually reviewed each participant’s data entry related to age, qualification(s), and year(s) qualifications obtained; work setting(s), years of clinical experience and their primary role in podiatry, to ensure that each entry was unique. All survey fields within sections were made mandatory to prevent missing data within sections. Participants who did not complete all five sections of the survey were excluded from the study.

### Statistical analysis

Data were imported and analysed using SPSS Version 29 (IBM Corporation, Armonk, NY, USA). Data were presented using descriptive statistics (mean [standard deviation; SD], or number [percentage]), as appropriate. Data substitution was not used for any missing data.

## Results

### Participant characteristics

Australian registered podiatrists (n = 103) completed the survey between November 2022 to July 2023. Sample characteristics are summarised in Table 1. Participants were distributed across all Australian states and territories, with the majority from Victoria (34%) and New South Wales (29%). The gender distribution was similar (54 women and 49 men). The mean age (standard deviation, SD) of participants was 40 (11) years, ranging from 24 to 69 years, with the most common age range being 30 to 39 years. The median (inter-quartile range) time taken to complete the survey was 13.5 (9.0 to 20.5) minutes.

**Table 1:** Participant characteristics (*n* = 103). Values are expressed as number (%) unless reported otherwise, where (%) indicates the proportion of all participants

By state or territory	
Australian Capital Territory	1 (1.0)
New South Wales	30 (29.1)
Northern Territory	1 (1.0)
Queensland	14 (13.6)
South Australia	8 (7.8)
Tasmania	4 (3.9)
Victoria	35 (34.0)
Western Australia	10 (9.7)
Gender, women	54 (52.4)
Age (years), mean (SD), range	39.6 (10.9), 24 to 69
Age	
29 years or younger	21 (20.4)
30 to 39 years	38 (36.9)
40 to 49 years	19 (18.4)
50 to 59 years	21 (20.4)
60 to 69 years	4 (3.9)
Year of qualification	
2021 to 2023	4 (3.9)
2011 to 2020	47 (45.6)
2001 to 2010	19 (18.4)
1991 to 2000	22 (21.4)
1981 to 1990	9 (8.7)
Before 1981	2 (1.9)
Educational institution podiatry qualifications obtained from	
Central Queensland University	2 (1.9)
Charles Stuart University	4 (3.9)
La Trobe University (previously Lincoln Institute of Health Sciences)	38 (36.9)
University of Newcastle Australia	6 (5.8)
Queensland University of Technology	11 (10.7)
University of South Australia (previously South Australian Institute of Technology)	10 (9.7)
Western Sydney University (previously University of Western Sydney)	8 (7.8)
Sydney Technical College (Sydney Institute of Technology or Technical and Further Education, New South Wales)	6 (5.8)
International (United Kingdom)	4 (3.9)
International (New Zealand)	2 (1.9)
International (South Africa)	1 (1.0)
Work setting	
Exclusively public	4 (3.9)
Exclusively private	90 (87.4)
Combination	9 (8.7)
Clinical years of experience, mean (SD), range	14.6 (11.2), 1 to 42
≤ 5 years	31 (30.1)
> 5 to 10 years	22 (21.4)
> 10 to 20 years	18 (17.5)
> 20 to 30 years	24 (23.3)
> 30 years	8 (7.8)
Principal role	
Clinician	93 (90.3)
Administrator	2 (1.9)
Teacher or educator	7 (6.8)
Researcher	1 (1.0)
Other	1 (1.0)
Frequency treating patients with midfoot OA	
Infrequently (at most 1 in the last 6 months)	2 (1.9)
Somewhat frequently (2 to 6 in the last 6 months)	37 (35.9)
Frequently (7 to 24 in the last 6 months)	43 (41.7)
Very frequently (> 24 in the last 6 months)	21 (20.4)
Completed postgraduate training in osteoarthritis	37 (36.0)
Day or weekend course(s)	22 (21.4)
Non-university course(s) or module(s)	8 (7.8)
University postgraduate	14 (13.6)
Other	4 (3.9)
Endorsed for prescribing scheduled medicines	5 (4.9)

*SD* standard deviation

A high proportion of participants (46%) graduated between 2011 and 2020. On average, they had 15 (11) years of clinical experience. The majority (87%) worked exclusively in private practice, while 9% worked in both private and public settings, and 4% worked exclusively in public settings. Few (5%) were endorsed for prescribing scheduled medicines. Forty-three participants (42%) reported treating patients with midfoot OA frequently (at least 7 to 24 patients in the last 6 months). Over a third of participants (36%) reported that they had completed further professional development in OA, with most opting for day or weekend courses (21%) or university postgraduate education (14%).

### Diagnostic approaches

Table 2 presents diagnostic approaches reported by participants. Most participants (96%) indicated that they would typically diagnose midfoot OA based on physical assessment (defined as history taking and musculoskeletal assessment).

**Table 2:** Diagnostic approaches reported by participants for a typical patient with midfoot OA

Physical assessment (history taking and musculoskeletal assessment)	99 (96.1)
Patient reported outcome measures	75 (72.8)
Numerical rating scale	31 (30.1)
Visual analogue scale	51 (49.5)
Foot Health Status Questionnaire	9 (8.7)
Manchester Foot Pain and Disability Index	8 (7.8)
Other	5 (4.9)
Self-reported site of foot pain	
Plantar forefoot	5 (4.9)
Plantar heel	2 (1.9)
Plantar medial arch	49 (47.6)
Plantar central midfoot	46 (44.7)
Plantar lateral midfoot	45 (43.7)
Dorsal metatarsals	11 (10.7)
Dorsal medial midfoot	100 (97.1)
Dorsal central midfoot	101 (98.1)
Dorsal lateral midfoot	87 (84.5)
Antero-medial ankle	29 (28.2)
Antero-lateral ankle	19 (18.4)
Anterior ankle	27 (26.2)
Posterior ankle	0 (0.0)
Musculoskeletal assessment	
Visual inspection	92 (89.3)
Passive movement testing	98 (95.1)
Resisted isometric muscle testing	61 (59.2)
Active movement testing	80 (77.7)
Palpation	99 (96.1)
Other	15 (14.6)
Special tests	23 (22.3)
Mixed comments with themes of assessing passive range of motion testing, imaging, resisted muscle isometric testing and performing calf raises	
Medical imaging	93 (90.3)
X-ray	64 (62.1)
Ultrasound	27 (26.2)
9 out of 27 participants (33.3%) use point-of-care ultrasound	
Magnetic resonance imaging (MRI)	14 (13.6)
Computed tomography (CT)	2 (1.9)
Bone scan	3 (2.9)
Other	2 (1.9)
Refer to other medical practitioners for further investigations	6 (5.8)
For blood tests, bone density scans or MRI	

Values are expressed as number (%), where (%) indicates the proportion of all participants

**Fig. 2:**
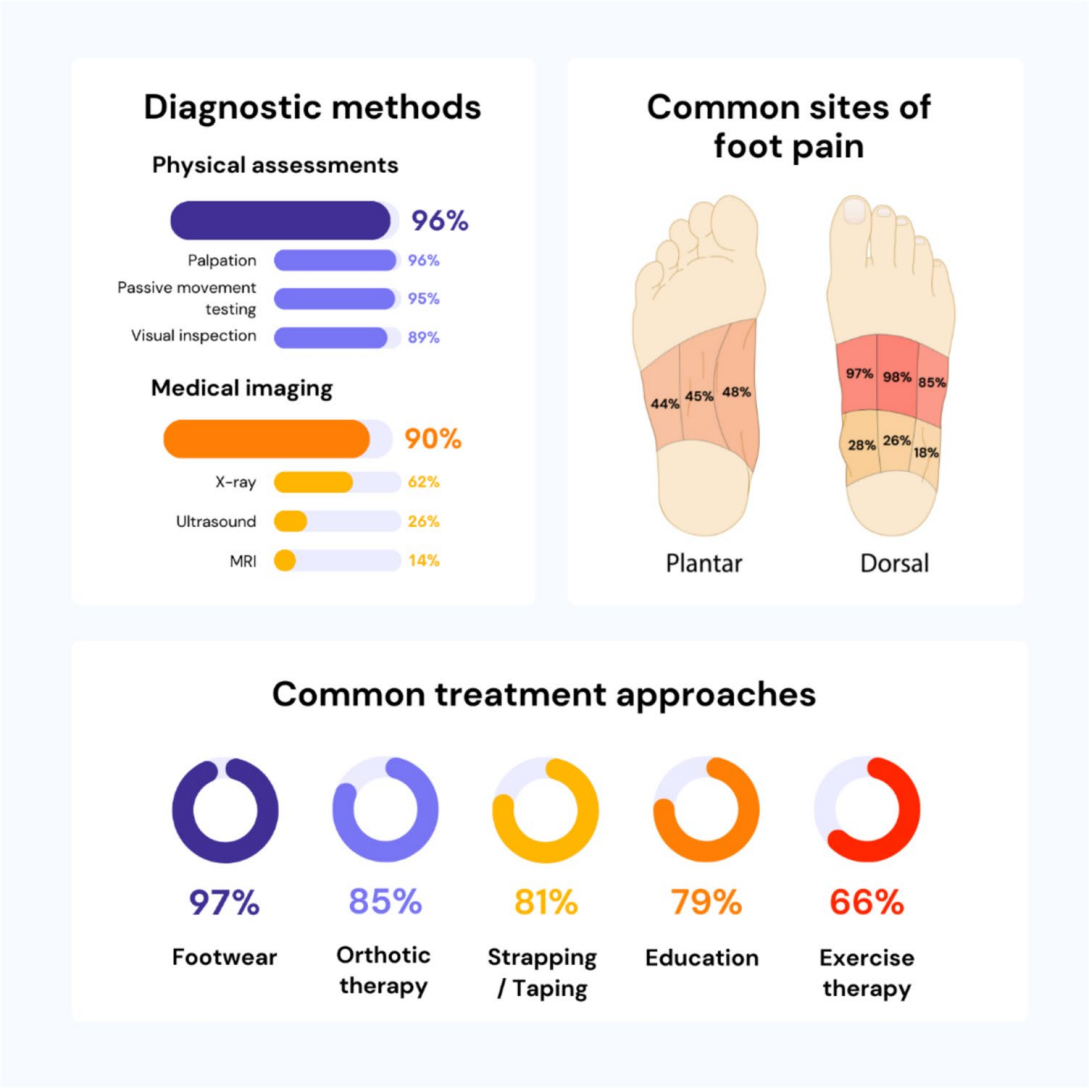
Midfoot OA assessment and management by podiatrists in Australia: key findings

Many participants (73%) indicated that they would incorporate a patient-reported outcome measure into the diagnostic assessment, with the most common being a visual analogue scale (50%) or numerical rating scale of pain severity (30%). Overall, almost all participants would expect a person with midfoot OA to typically report pain in the dorsal area of the medial (97%) and central (98%) midfoot, followed by the dorso-lateral aspect (85%) (Fig. 2). Approximately half (44 to 48%) reported that they would anticipate pain in the plantar medial arch, plantar central midfoot, and plantar lateral midfoot. Approximately one quarter (26 to 28%) indicated that they would expect pain in the antero-medial ankle and anterior ankle regions.

When conducting musculoskeletal assessment to aid in diagnosis, the most common approaches reported were palpation (96%), passive movement testing (95%), visual inspection (89%), active movement testing (78%), and resisted isometric muscle testing (59%). Additionally, 23 (22%) participants reported using ‘special tests’ to diagnose midfoot OA. These tests varied and included different passive movement and resisted isometric tests around the midfoot joints, and other active movement tests such as calf raises.

Additionally, 93 (90%) participants reported using radiological imaging to assist in their diagnosis. Among imaging modalities, plain film x-ray (62%), ultrasound (26%), and MRI (14%) were most used (Fig. 2). Of the 27 participants who reported using ultrasound, 9 (33%) reported that they would perform point-of-care ultrasound. Six participants (6%) reported that they would refer the patient to another medical practitioner, such as general practitioners or rheumatologists, or for further investigations such as blood tests, bone density scans and MRI.

### Assessment approaches

Table 3 presents the participants’ approaches to assess associated factors, physical impairments, and comorbidities. Nearly all participants (98%) indicated that they would assess the range of motion of the foot and ankle joints (ankle joint: 93%; midfoot joints: 96%; first metatarsophalangeal joint: 94%). Few participants reported performing range of motion assessments of the hip (12%) or knee (6%). Observing static alignment (71%), which included assessing foot posture (69%), was common. Of those who would evaluate muscle strength (72%), the majority would test ankle plantarflexors (70%), ankle dorsiflexors (68%), ankle invertors (68%), and ankle evertors (66%). Approximately half of participants would evaluate the hallux plantarflexors (51%) and lesser toe plantarflexors (46%).

**Table 3:** Assessment approaches reported by participants to identify associated factors, physical impairments, and comorbidities for a typical patient with midfoot OA

Range of motion	101 (98.1)
Ankle joint	96 (93.2)
Midfoot joint	99 (96.1)
First metatarsophalangeal joint	97 (94.2)
Other	31 (30.1)
Knee joint	6 (5.8)
Hip joint	12 (11.7)
Static alignment	73 (70.9)
Foot posture	71 (68.9)
Other	19 (18.4)
Knee	11 (10.7)
Muscle strength	74 (71.8)
Ankle plantarflexors	72 (69.9)
Ankle dorsiflexors	70 (68.0)
Ankle invertors	70 (68.0)
Ankle evertors	68 (66.0)
Lesser toe plantarflexors	47 (45.6)
Hallux plantarflexors	52 (50.5)
Other	5 (4.9)
Gait analysis	98 (95.1)
Visual	97 (94.2)
Computerised kinematics	15 (14.6)
Computerised kinetics	14 (28.2)
Plantar pressures	29 (28.2)
Other	7 (6.8)
Functional tests	85 (82.5)
Calf raises	78 (75.7)
Hops	24 (23.3)
Stairs	24 (23.3)
Balance test	64 (62.1)
Other	12 (11.7)
Squats	9 (8.7)
Other	2 (1.9)
Body composition assessment	68 (66.0)
Observation	57 (55.3)
Measure waist/hip circumference	1 (1.0)
Ask the patient directly	22 (21.4)
Calculate body mass index	14 (13.6)
Measure patient’s weight using scales	10 (9.7)
Other	4 (3.9)
Comorbidities assessment	96 (93.2)
Cognitive/psychological assessment	64 (62.1)

Values are expressed as number (%), where (%) indicates the proportion of all participants

Most participants (95%) reported that they would conduct gait analysis, commonly using visual gait analysis (94%) and occasionally utilising computerised analysis of kinematics (15%), kinetics (28%), and plantar pressure analysis (28%). Functional tests would be used by 83% of participants, with calf raises (83%) and balance tests (62%) being the most common. Some participants would also perform assessments such as hopping (23%), stair climbing (23%), and less frequently, squats (8%).

Approximately two-thirds (66%) of participants reported that they would assess the patient’s body composition. This assessment included observation (55%), directly questioning about weight (21%), calculating body mass index (14%), measurement of weight using scales (10%), and measuring waist and hip circumference (1%). Additionally, most participants (93%) indicated that they would further assess for the presence of comorbidities, with more than half (62%) assessing the cognitive and psychological status of the patient.

### Treatment approaches

Table 4 summarises treatment strategies that participants would use to manage midfoot OA. Overall, the most common approaches were addressing footwear (97%), prescribing foot orthoses (85%), using strapping/taping (81%), providing education (79%), and providing exercise therapy (75%) (Fig. 2).

**Table 4:** Treatment approaches reported by participants for a typical patient with midfoot OA

Cryotherapy (cold therapy)	10 (9.7)
Dry needling	20 (19.4)
Education	81 (78.6)
Extracorporeal shock wave therapy	13 (12.6)
Exercise therapy (including advice, strengthening, range of motion, aerobic exercise)	77 (74.8)
Foot and ankle extrinsic muscle strengthening	68 (66.0)
Intrinsic foot muscle strengthening	58 (56.3)
Modification of physical activity	50 (48.5)
Alternative exercises (e.g. general advice around low impact exercise such as swimming)	47 (45.6)
Avoidance of aggravating activity	41 (39.8)
Increasing activity	13 (12.6)
Pacing of activities (to do a little bit often)	33 (32.0)
Reducing activity	15 (14.6)
Other(s)	1 (1.0)
Proprioception/balance	40 (38.8)
Self-mobilisation	28 (27.2)
Self-myofascial release (e.g. foam rolling)	21 (20.4)
Stretching	45 (43.7)
Refer to others for exercise therapy	21 (20.4)
Refer to exercise physiology	14
Refer to physiotherapy	10
Other	8 (7.8)
Foot mobilisation and manipulation therapy	45 (43.7)
Footwear	100 (97.1)
Adequate length	72 (69.9)
Adequate width	82 (79.6)
Adequate depth	90 (87.4)
Adequate fixation	60 (58.3)
Rigid heel counter	64 (62.1)
Rigid midsole	60 (58.3)
Rocker bottom outsole	72 (69.9)
Minimalist ‘barefoot’	2 (1.9)
Cushioned	42 (40.8)
Other	10 (9.7)
Shoe lacing adjustments	4 (3.9)
Gait retraining	12 (11.7)
Laser therapy	10 (9.7)
Massage therapy	22 (21.4)
Orthotic therapy	88 (85.4)
Shoe stiffening inserts	31 (30.1)
Arch contouring foot orthoses	84 (81.6)
Arch contouring prefabricated foot orthoses	41 (39.8)
29 out of 41 participants (71%) would modify a prefabricated device	
Arch contouring custom-made foot orthoses	66 (64.1)
Padding	44 (42.7)
Pharmacological therapy (including topical and oral analgesics, injection therapy and, complementary therapies and nutraceuticals)	43 (41.7)
Topical therapies	27 (26.2)
NSAIDs	27 (26.2)
Capsaicin	2 (1.9)
Other	2 (1.9)
Fisiocrem®, Pain Away®, Nervoderm®	
Oral analgesics	33 (32.0)
Paracetamol	20 (19.4)
NSAIDs	29 (28.2)
Other	4 (3.9)
Consult with General practitioner, follow hip knee OA RACGP guidelines	
Injection therapy	16 (15.5)
Corticosteroid	14 (13.6)
Other	2 (1.9)
Prolotherapy/plasma-rich-plasma injections	
Perform injection themselves	4 (3.9)
Refer injection therapy to others	
Foot and ankle orthopaedic surgeon	3 (2.9)
General practitioner	2 (1.9
Podiatric surgeon	2 (1.9)
Radiologist	11 (10.7)
Sports physician	6 (5.8)
Endorsed podiatrist	1 (1.0)
Complementary therapies and nutraceuticals	6 (5.8)
Glucosamine	4 (3.9)
Chondroitin	1 (1.0)
Turmeric/curcumin	4 (3.9)
Other Vitamin D/K2, New Zealand green lip muscle extract, patient’s preference	3 (2.9)
Strapping/taping	83 (80.6)
Low dye	72 (69.9)
High dye	13 (12.6)
‘J’ strap	12 (11.7)
Other	12 (11.7)
Compression, combine with kinesiology tape	
Rigid tape	79 (76.7)
Kinesiology tape	12 (12.6)
Other	2 (1.9)
Thermotherapy (heat therapy)	14 (13.6)
Trigger point techniques	14 (13.6)
Other	14 (13.6)
Address weight management	39 (37.9)
Provide advice about weight loss	17 (16.5)
Actively deliver a weight loss program	0 (0.0)
Refer to a dietitian	16 (15.5)
Refer on to a weight loss program	10 (9.7)
Other	13 (12.6)
Refer to other healthcare practitioners for co-management in the initial six weeks	23 (22.3)
Acupuncturist	0 (0.0)
Chiropractor	1 (1.0)
Dietitian	7 (6.8)
Exercise physiologist	15 (14.6)
General practitioner	10 (9.7)
Myotherapist	0 (0.0)
Occupational therapist	2 (1.9)
Other podiatrist(s)	0 (0.0)
Orthopaedic surgeon	4 (3.9)
Osteopath	2 (1.9)
Pain clinic	3 (2.9)
Pharmacist	5 (4.9)
Physiotherapist	15 (14.6)
Podiatric surgeon	2 (1.9)
Rheumatologist	7 (6.8)
Sports physician	3 (2.9)
Other	1 (1.0)

Values are expressed as number (%), where (%) indicates the proportion of all participants

*RACGP* Royal Australian College of General Practitioners

*NSAIDs* Non-steroidal anti-inflammatory drugs

#### Footwear advice

Common footwear advice included ensuring adequate length (70%), width (80%), and depth (87%). Additional footwear recommendations included a rocker bottom outsole (70%), a rigid heel counter (62%), a rigid midsole (58%), adequate fixation (58%), and a cushioned outsole (41%). Minimalist footwear (2%) was rarely recommended. Four participants (4%) would recommend adjustments to shoe lacing.

#### Strapping or taping

Strapping or taping using a low Dye technique was commonly reported (70%). High Dye (13%) and J-strap taping (12%) would be used less frequently. Additional comments (12%) included the use of compression or a combination of taping techniques involving both rigid and kinesiology tape. Further, most participants preferred rigid tape (77%) over kinesiology tape (13%).

#### Orthotic therapy

Most participants (85%) indicated that they would provide arch contouring foot orthoses and/or shoe stiffening inserts. A high proportion of participants (82%) indicated that they would use arch contouring foot orthoses and 30% would use shoe stiffening inserts (participants were given the option to select multiple orthotic styles). Sixty-six participants (64%) indicated that they would provide a custom-made arch contouring foot orthosis, and 41 participants (40%) indicated that they would provide a prefabricated arch contouring foot orthosis. Out of the participants who would use prefabricated arch contouring foot orthoses, more than two-thirds (29 out of 41 participants [71%]) reported that they would modify these prior to dispensing.

#### Exercise therapy

Exercise therapy primarily included advice regarding physical activity, strengthening, improving range of motion and physical activity. Many participants (66%) specified that they would provide extrinsic foot and ankle strengthening exercises, followed by intrinsic foot muscle strengthening exercises (56%). Nearly half (49%) reported that they would provide advice regarding modification of physical activity. Of these, 47 participants (46%) specified that they would provide general advice on alternative low impact exercises such as swimming, and 41 (40%) indicated that they would advise avoiding aggravating activities. Around one-third of participants (32%) reported that they would advise pacing of activities (i.e., ‘to do a little bit often’) and a smaller number indicated that they would advise either reducing (15%) or increasing activity (13%) depending on what they considered appropriate. Other exercise therapy approaches included stretching (44%), improving proprioception or balance (39%), self-mobilisation (27%), and self-myofascial release exercises (20%). One-fifth (20%) reported that they would refer to another healthcare practitioner, such as exercise physiologists or physiotherapists for exercise therapy.

#### Weight management

More than one-third of participants (38%) reported that they would address weight management. This included providing advice about weight loss (17%), referring to a dietitian (16%), and referring to a weight loss program (10%). None of the participants specified that they would actively deliver a weight loss program.

#### Co-management with other healthcare professionals

Twenty-three participants (22%) indicated that they would refer to other healthcare professionals during the initial six weeks. The most common healthcare professionals for referral included exercise physiologists (15%), physiotherapists (15%), general practitioners (10%), dietitians (7%) and rheumatologists (7%).

### Treatment timeline

The anticipated timeline for managing midfoot OA is presented in Table 5. Over half of the participants (55%) specified that they would assess and treat the patient at least three to four times for their midfoot pain over a period of five to six weeks (26%) and expect a ‘satisfactory’ treatment response within one to two months (34%). Some others (23%) would expect treatment duration to take more than 10 weeks. Participants indicated that they would consider referring this patient to another healthcare practitioner if there was no ‘satisfactory’ treatment response between two to three months (36%). The most mentioned healthcare practitioner for initial referral was the patient’s general practitioner (30%), followed by a podiatric surgeon (18%), and an orthopaedic surgeon (15%).

**Table 5:** Treatment expectations and timeline for a typical patient with midfoot OA

Number of times participants would typically see the patient for midfoot pain	
Once	4 (3.9)
Twice	8 (7.8)
3 to 4 times	57 (55.3)
5 to 8 times	25 (24.3)
> 8 times	9 (8.7)
Treatment duration	
1 to 2 weeks	8 (7.8)
3 to 4 weeks	12 (11.7)
5 to 6 weeks	27 (26.2)
7 to 8 weeks	22 (21.4)
9 to 10 weeks	10 (9.7)
> 10 weeks	24 (23.3)
Expected treatment timeframe	
Within first week	9 (8.7)
Between 1 week and 1 month	29 (28.2)
Between 1 and 2 months	35 (34.0)
Between 2 and 3 months	23 (22.3)
Between 3 and 6 months	6 (5.8)
Between 6 and 12 months	1 (1.0)
> 12 months	0 (0.0)
Time points to consider referring to another healthcare practitioner	
Within first week	1 (1.0)
Between 1 week and 1 month	12 (11.7)
Between 1 and 2 months	28 (27.2)
Between 2 and 3 months	37 (35.9)
Between 3 and 6 months	20 (19.4)
Between 6 and 12 months	5 (4.9)
> 12 months	0 (0.0)
Never	0 (0.0)
Most common healthcare practitioner referred to if not improving as expected	
General practitioner	31 (30.1)
Podiatric surgeon	18 (17.5)
Orthopaedic surgeon	15 (14.6)

Values are expressed as number (%), where (%) indicates the proportion of all participants

## Discussion

This study is the first to investigate the current practices of Australian podiatrists in assessing and managing midfoot OA. Our findings show that history taking, musculoskeletal assessments and medical imaging were commonly used methods for diagnosing midfoot OA. Almost all participants would also include assessment of range of motion of the foot joints and gait analysis, and the majority would include functional tests, assessment of muscle strength, static foot posture, comorbidities and body composition in their assessment approaches. The most common treatment approaches were footwear advice, strapping/taping, orthotic therapy, education and exercise advice. Participants generally expect a ‘satisfactory’ treatment response within one to two months and would typically see a patient with midfoot OA three to four times. Participants indicated that they would consider referral to other healthcare professionals if the condition did not respond favourably to usual treatment approaches within two to three months.

Many participants indicated that they would use patient-reported outcome measures to ascertain pain severity as part of their diagnostic assessment, which is in line with clinical guidelines [45, 46]. Consistent with previous studies [6, 8, 9], dorsally located midfoot pain was the most frequently reported site of foot pain in people with midfoot OA. Palpation or observation of a dorsal midfoot exostosis was commonly reported, however its association as a clinical feature of midfoot OA remains uncertain [6, 7]. The common use of passive movement testing is consistent with evidence that passive movement testing of individual tarsometatarsal joints may be a valid test for symptomatic tarsometatarsal joints [47].

Most participants would incorporate medical imaging into their approach to diagnose midfoot OA, with the majority utilising plain film x-ray. This finding is consistent with a previous survey of podiatrists and physical therapists regarding the diagnosis of first metatarsophalangeal joint OA [42], and a previous review indicating that x-ray imaging is most frequently used in foot OA research [48]. Notably, our study demonstrated that approximately 26% of participants use musculoskeletal ultrasound to assess midfoot OA, a higher proportion compared to the use of ultrasound for diagnosing first metatarsophalangeal joint OA [42]. Furthermore, a third of these participants used ultrasound at point-of-care, suggesting a relatively high uptake of diagnostic ultrasound amongst podiatrists. This prevalent use of radiological imaging contrasts with international clinical guidelines for knee and hip OA, which advise against the routine use of imaging for OA diagnosis [45, 46]. This discrepancy may stem from the current lack of evidence supporting the diagnostic accuracy of clinical tests for midfoot OA [6, 7]. Additionally, the complex and intricate anatomy of the midfoot poses challenges in conducting musculoskeletal assessments that can accurately evaluate each specific structure of the midfoot [5]. These findings highlight the need for further research investigating clinical non-radiological assessments for midfoot OA that can demonstrate high diagnostic accuracy.

Several assessment approaches were reported to be used to identify associated factors and impairments in midfoot OA. Nearly all participants would perform a dynamic gait analysis, with the majority using visual analysis. Over a quarter also used computerised kinetics or plantar pressure analysis. Previous research shows that people with midfoot OA have increased peak plantar pressures, pressure time integrals and contact times in the heel and midfoot [15], and exhibit greater forces and pressures within the midfoot during walking and increased lateral forefoot forces and pressure during toe-off [49]. These biomechanical alterations support the use of objective gait analysis and additional computerised software, which may be useful in clinical practice to aid decision-making in relation to treatment. Most participants reported that they would use functional tests, such as calf raises and balance tests, despite the limited evidence to support the use of such tests other than difficulties with step descent [12]. Many participants would also assess foot and ankle muscle strength, as well as perform a static foot posture assessment. The assessment of these physical impairments is consistent with evidence that people with midfoot OA have reduced foot and leg muscle strength [3], and are more likely to have a flatter foot posture [6, 7, 15, 17]. More research is needed to validate these assessment approaches, so that their associated findings may be considered when formulating intervention strategies for midfoot OA.

Most participants indicated that they would assess for comorbidities and body composition. This is consistent with evidence that people with symptomatic midfoot OA are more likely to be obese and report non-musculoskeletal comorbidities [4]. Associations between body mass index and the severity of foot pain and foot-related disability have been found in people with first metatarsophalangeal joint OA [50], and further investigation is needed to determine whether similar associations exist for midfoot OA. If confirmed, this would support the inclusion of weight management as a core treatment, as recommended in OA clinical guidelines [45].

A range of non-surgical treatment approaches would commonly be used by podiatrists to manage midfoot OA. Most participants reported that they would provide education, including information and support about understanding OA, engaging in physical activity, pacing of activities and weight management. The provision of education and exercise advice is consistent with recommendations from OA guidelines, where education and exercise advice are considered ‘core’ treatments [45, 46]. Given that foot and ankle muscle strength is often reduced in people with midfoot OA [3], this further supports the use of exercise for this condition. More research is needed to evaluate the effectiveness of exercise interventions for midfoot OA.

A key finding from this study was the widespread use of mechanical interventions by podiatrists to treat midfoot OA. Footwear with extra depth and width, as well as rocker-bottom footwear, were frequently recommended for midfoot OA. Rocker-bottom shoes, designed with a curved outsole to facilitate propulsion, appear to be a feasible intervention. However, the evidence supporting footwear modification is limited to a single case-series study [32], so further research is required to determine the effectiveness of footwear interventions in midfoot OA. Regarding orthotic therapy, most participants indicated that they would use arch contouring foot orthoses and/or shoe stiffening inserts. There is some evidence demonstrating effectiveness of these specific types of orthotic devices for midfoot OA. However, the current evidence is limited to one feasibility trial [8] and three case-series trials [9, 31, 32], so there is uncertainty regarding the effectiveness of these interventions. Indeed, the lack of rigorous randomised trials investigating footwear and orthotic devices for midfoot OA is consistent with NICE guidelines [45] and international working group recommendations [5] that more research is needed to determine the effectiveness of footwear and orthotic devices for foot OA.

There is currently very limited evidence regarding the assessment and management of midfoot OA. Previous studies have reported the clinical characteristics of midfoot OA [3, 6, 7] but did not report how the condition was diagnosed. Further, although a range of non-surgical interventions [8, 9, 22, 31–33] have been recommended, our recent systematic review [35] demonstrated that the evidence for effectiveness of these interventions for the management of midfoot OA is limited. This study represents an important initial step in identifying usual practices of podiatrists who assess and manage people with midfoot OA, which should provide valuable guidance for clinicians and researchers. It will help in designing research studies that align with current clinical practices. Strengths of the study include the proportionate representation of participants in terms of gender, age, workplace settings (private and public) across Australia [41, 51]. Further, we obtained detailed information regarding the assessment and management cycle by podiatrists including expectations for the timing of treatment responses. It is noteworthy that the concept of co-management with other healthcare providers was reported as early as the first six weeks. This suggests that managing midfoot OA often requires a multidisciplinary approach, likely due to the complexity of the impairments and comorbidities associated with the condition. People with midfoot OA may experience better outcomes through comprehensive, collaborative care, rather than relying on treatment from a single healthcare profession.

Despite the strengths of this study, there are several limitations that need to be considered. Firstly, despite our efforts to ensure a proportionately representative sample across Australia, the survey had a higher response rate from podiatrists in Victoria and New South Wales, with less representation from Queensland. Nevertheless, the overall distribution of responses was consistent with the podiatrist population across Australia [41]. Secondly, all participants were podiatrists, and few had endorsement for scheduled medicines, so the findings may not be generalisable to other medical or healthcare professions who manage midfoot OA, or podiatrists with a more advanced scope of practice. The findings are also specific to podiatric practice in Australia and may have limited applicability in countries where such practices do not exist. Thirdly, although research has shown that case vignettes are valid tools for assessing clinical practices [43, 44], it is possible that clinicians may have provided responses based on the vignette that may not reflect their usual approaches. Lastly, it is important to emphasise that this study is a survey of healthcare professionals (i.e., podiatrists), rather than an evidence-based clinical guideline. The results reflect the current clinical practice of Australian podiatrists who report treating at least 2 to 24 people with midfoot OA over half a year. This study does not provide guidance as to what is best practice but rather offers insights to common practices for the assessment and non-surgical management of midfoot OA.

## Conclusion

Australian podiatrists use a range of approaches for the assessment and non-surgical management of midfoot OA. Podiatrists most commonly use history taking, musculoskeletal assessments including palpation and passive movement testing; and x-ray imaging to diagnose midfoot OA. Several assessment approaches are used to identify associated factors and impairments including muscle strength and functional tests, comorbidities, and body composition. Podiatrists frequently use education including footwear advice, orthotic therapy, strapping/taping, and exercise therapy to manage midfoot OA. These findings provide insights into common practices for assessing and managing midfoot OA and have the potential to inform the design of future studies in developing valid assessment approaches and effective interventions for this condition.

## Supplementary Information

Below is the link to the electronic supplementary material.

Supplementary file1 (PDF 1092 KB)

Supplementary file2 (DOCX 22 KB)

Supplementary file3 (DOCX 22 KB)

## Data Availability

Data from this study is available from the corresponding author upon reasonable request, in compliance with institutional policies and ethical guidelines.

## References

[1] Roddy E, Thomas MJ, Marshall M et al (2015) The population prevalence of symptomatic radiographic foot osteoarthritis in community-dwelling older adults: cross-sectional findings from the clinical assessment study of the foot. Ann Rheum Dis 74:156–163. 10.1136/annrheumdis-2013-203804 24255544 10.1136/annrheumdis-2013-203804 PMC4283621

[2] Rao S, Baumhauer JF, Nawoczenski DA (2011) Is barefoot regional plantar loading related to self-reported foot pain in patients with midfoot osteoarthritis. Osteoarthr Cartil 19:1019–1025. 10.1016/j.joca.2011.04.006 10.1016/j.joca.2011.04.006 21571084

[3] Arnold JB, Halstead J, Grainger AJ, Keenan A-M, Hill CL, Redmond Anthony C (2021) Foot and leg muscle weakness in people with midfoot osteoarthritis. Arthritis Care Res (Hoboken) 73:772–780. 10.1002/acr.24182 32170831 10.1002/acr.24182

[4] Thomas MJ, Peat G, Rathod T et al (2015) The epidemiology of symptomatic midfoot osteoarthritis in community-dwelling older adults: cross-sectional findings from the clinical assessment study of the foot. Arthritis Res Ther 17:178. 10.1186/s13075-015-0693-3 26166410 10.1186/s13075-015-0693-3 PMC4499901

[5] Arnold JB, Bowen CJ, Chapman LS et al (2022) International Foot and Ankle Osteoarthritis Consortium review and research agenda for diagnosis, epidemiology, burden, outcome assessment and treatment. Osteoarthr Cartil 30:945–955. 10.1016/j.joca.2022.02.603 10.1016/j.joca.2022.02.603 PMC10464637 35176480

[6] Arnold JB, Marshall M, Thomas MJ, Redmond AC, Menz HB, Roddy E (2019) Midfoot osteoarthritis: potential phenotypes and their associations with demographic, symptomatic and clinical characteristics. Osteoarthr Cartil 27:659–666. 10.1016/j.joca.2018.12.022 10.1016/j.joca.2018.12.022 30660723

[7] Thomas MJ, Roddy E, Rathod T et al (2015) Clinical diagnosis of symptomatic midfoot osteoarthritis: cross-sectional findings from the clinical assessment study of the foot. Osteoarthr Cartil 23:2094–2101. 10.1016/j.joca.2015.06.010 10.1016/j.joca.2015.06.010 PMC4672469 26093213

[8] Halstead J, Chapman GJ, Gray JC et al (2016) Foot orthoses in the treatment of symptomatic midfoot osteoarthritis using clinical and biomechanical outcomes: a randomised feasibility study. Clin Rheumatol 35:987–996. 10.1007/s10067-015-2946-6 25917211 10.1007/s10067-015-2946-6 PMC4819552

[9] Rao S, Baumhauer JF, Becica L, Nawoczenski DA (2009) Shoe inserts alter plantar loading and function in patients with midfoot arthritis. J Orthop Sports Phys Ther 39:522–531. 10.2519/jospt.2009.2900 19574663 10.2519/jospt.2009.2900

[10] Kurup H, Vasukutty N (2020) Midfoot arthritis-current concepts review. J Clin Orthop Trauma 11:399–405. 10.1016/j.jcot.2020.03.002 32405198 10.1016/j.jcot.2020.03.002 PMC7211829

[11] Patel A, Rao S, Nawoczenski D, Flemister AS, DiGiovanni B, Baumhauer JF (2010) Midfoot arthritis. J Am Acad Orthop Surg 18:417–425. 10.5435/00124635-201007000-00004 20595134 10.5435/00124635-201007000-00004

[12] Rao S, Baumhauer JF, Tome J, Nawoczenski DA (2009) Comparison of in vivo segmental foot motion during walking and step descent in patients with midfoot arthritis and matched asymptomatic control subjects. J Biomech 42:1054–1060. 10.1016/j.jbiomech.2009.02.006 19409567 10.1016/j.jbiomech.2009.02.006

[13] Gong Q, Halstead J, Keenan A-M, Milanese S, Redmond AC, Arnold JB (2023) Intrinsic foot muscle size and associations with strength, pain and foot-related disability in people with midfoot osteoarthritis. Clin Biomech (Bristol, Avon) 101:105865. 10.1016/j.clinbiomech.2022.105865 10.1016/j.clinbiomech.2022.105865 36565560

[14] Arnold JB, Halstead J, Martín-Hervás C et al (2023) Bone marrow lesions and MRI-detected structural abnormalities in people with midfoot pain and osteoarthritis: a cross-sectional study. Arthritis Care Res (Hoboken) 75:1113–1122. 10.1002/acr.24955 35593411 10.1002/acr.24955 PMC10952448

[15] Lithgow MJ, Munteanu SE, Buldt AK, Arnold JB, Kelly LA, Menz HB (2020) Foot structure and lower limb function in individuals with midfoot osteoarthritis: a systematic review. Osteoarthr Cartil 28:1514–1524. 10.1016/j.joca.2020.08.012 10.1016/j.joca.2020.08.012 32889086

[16] Menz HB, Munteanu SE, Zammit GV, Landorf KB (2010) Foot structure and function in older people with radiographic osteoarthritis of the medial midfoot. Osteoarthr Cartil 18:317–322. 10.1016/j.joca.2009.11.010 10.1016/j.joca.2009.11.010 19948268

[17] Lithgow MJ, Buldt AK, Munteanu SE et al (2024) Structural foot characteristics in people with midfoot osteoarthritis: cross-sectional findings from the clinical assessment study of the foot. Arthritis Care Res (Hoboken) 76:225–230. 10.1002/acr.25217 37563733 10.1002/acr.25217 PMC11497243

[18] Rathod T, Marshall M, Thomas MJ et al (2016) Investigations of potential phenotypes of foot osteoarthritis: cross-sectional analysis from the clinical assessment study of the foot. Arthritis Care Res (Hoboken) 68:217–227. 10.1002/acr.22677 26238801 10.1002/acr.22677 PMC4819686

[19] Menz HB, Munteanu SE, Landorf KB, Zammit GV, Cicuttini FM (2007) Radiographic classification of osteoarthritis in commonly affected joints of the foot. Osteoarthr Cartil 15:1333–1338. 10.1016/j.joca.2007.05.007 10.1016/j.joca.2007.05.007 17625925

[20] Reijnierse M, Griffith JF (2023) High-resolution ultrasound and MRI in the evaluation of the forefoot and midfoot. J Ultrason 23:e251–e271. 10.15557/jou.2023.0033 38020514 10.15557/jou.2023.0033 PMC10668940

[21] Omar IM, Weaver JS, Altbach MI et al (2023) Imaging of osteoarthritis from the ankle through the midfoot. Skelet Radiol 52:2239–2257. 10.1007/s00256-023-04287-7 10.1007/s00256-023-04287-7 PMC10400729 36737484

[22] Drakonaki EE, Kho JSB, Sharp RJ, Ostlere SJ (2011) Efficacy of ultrasound-guided steroid injections for pain management of midfoot joint degenerative disease. Skelet Radiol 40:1001–1006. 10.1007/s00256-010-1094-y 10.1007/s00256-010-1094-y 21274710

[23] Camerer M, Ehrenstein B, Hoffstetter P, Fleck M, Hartung W (2017) High-resolution ultrasound of the midfoot: sonography is more sensitive than conventional radiography in detection of osteophytes and erosions in inflammatory and non-inflammatory joint disease. Clin Rheumatol 36:2145–2149. 10.1007/s10067-017-3658-x 28478580 10.1007/s10067-017-3658-x

[24] Iagnocco A, Filippucci E, Riente L et al (2011) Ultrasound imaging for the rheumatologist XXXV. Sonographic assessment of the foot in patients with osteoarthritis. Clin Exp Rheumatol 29:757–762 22041178

[25] Steadman J, Sripanich Y, Rungprai C, Mills MK, Saltzman CL, Barg A (2021) Comparative assessment of midfoot osteoarthritis diagnostic sensitivity using weightbearing computed tomography vs weightbearing plain radiography. Eur J Radiol 134:109419. 10.1016/j.ejrad.2020.109419 33259992 10.1016/j.ejrad.2020.109419

[26] Verhoeven N, Vandeputte G (2012) Midfoot arthritis: diagnosis and treatment. Foot Ankle Surg 18:255–262. 10.1016/j.fas.2012.04.004 23093120 10.1016/j.fas.2012.04.004

[27] Nathan M, Mohan H, Vijayanathan S, Fogelman I, Gnanasegaran G (2012) The role of 99mTc-diphosphonate bone SPECT/CT in the ankle and foot. Nucl Med Commun 33:799–807 22692578 10.1097/MNM.0b013e328355880b

[28] Paterson KL, Gates L (2019) Clinical assessment and management of foot and ankle osteoarthritis: a review of current evidence and focus on pharmacological treatment. Drugs Aging 36:203–211. 10.1007/s40266-019-00639-y 30680680 10.1007/s40266-019-00639-y

[29] Paterson KL, Harrison C, Britt H, Hinman RS, Bennell KL (2018) Management of foot/ankle osteoarthritis by Australian general practitioners: an analysis of national patient-encounter records. Osteoarthr Cartil 26:888–894. 10.1016/j.joca.2018.03.013 10.1016/j.joca.2018.03.013 29656142

[30] Thomas MJ, Moore A, Roddy E, Peat G (2013) “Somebody to say ‘come on we can sort this’”: a qualitative study of primary care consultation among older adults with symptomatic foot osteoarthritis. Arthritis Care Res (Hoboken) 65:2051–2055. 10.1002/acr.22073 23861315 10.1002/acr.22073 PMC4225467

[31] Yi T, Kim JH, Oh-Park M, Hwang JH (2018) Effect of full-length carbon fiber insoles on lower limb kinetics in patients with midfoot osteoarthritis: a pilot study. Am J Phys Med Rehabil 97:192–199. 10.1097/PHM.0000000000000821 28914616 10.1097/PHM.0000000000000821

[32] Ibuki A, Cornoiu A, Clarke A, Unglik R, Beischer A (2010) The effect of orthotic treatment on midfoot osteoarthritis assessed using specifically designed patient evaluation questionnaires. Prosthet Orthot Int 34:461–471. 10.3109/03093646.2010.503672 20977387 10.3109/03093646.2010.503672

[33] Protheroe D, Gadgil A (2018) Guided intra-articular corticosteroid injections in the midfoot. Foot Ankle Int 39:1001–1004. 10.1177/1071100718779983 29936864 10.1177/1071100718779983

[34] Halstead J, Munteanu SE (2023) Current and future advances in practice: mechanical foot pain. Rheumatol Adv Pract 7:rkad081. 10.1093/rap/rkad081 38091412 10.1093/rap/rkad081 PMC10712443

[35] Lim PQX, Lithgow MJ, Kaminski MR, Landorf KB, Menz HB, Munteanu SE (2023) Efficacy of non-surgical interventions for midfoot osteoarthritis: a systematic review. Rheumatol Int 43:1409–1422. 10.1007/s00296-023-05324-3 37093273 10.1007/s00296-023-05324-3 PMC10261166

[36] Sharma A, Minh Duc NT, Luu Lam Thang T et al (2021) A consensus-based checklist for reporting of survey studies (CROSS). J Gen Intern Med 36:3179–3187. 10.1007/s11606-021-06737-1 33886027 10.1007/s11606-021-06737-1 PMC8481359

[37] Zimba O, Gasparyan AY (2023) Designing, conducting, and reporting survey studies: a primer for researchers. J Korean Med Sci 38:e403. 10.3346/jkms.2023.38.e403 38084027 10.3346/jkms.2023.38.e403 PMC10713437

[38] Harris PA, Taylor R, Minor BL et al (2019) The REDCap consortium: building an international community of software platform partners. J Biomed Inform 95:103208. 10.1016/j.jbi.2019.103208 31078660 10.1016/j.jbi.2019.103208 PMC7254481

[39] Harris PA, Taylor R, Thielke R, Payne J, Gonzalez N, Conde JG (2009) Research electronic data capture (REDCap)—a metadata-driven methodology and workflow process for providing translational research informatics support. J Biomed Inform 42:377–381. 10.1016/j.jbi.2008.08.010 18929686 10.1016/j.jbi.2008.08.010 PMC2700030

[40] Sample size calculator (2022). https://www.surveymonkey.com/mp/sample-size-calculator/. Accessed 20 Oct 2022

[41] Podiatry Board of Australia: podiatry registrant data (2024). https://www.podiatryboard.gov.au/About/Statistics.aspx. Accessed 10 Dec 2024

[42] Paterson KL, Hinman RS, Menz HB, Bennell KL (2020) Management of first metatarsophalangeal joint osteoarthritis by physical therapists and podiatrists in Australia and the United Kingdom: a cross-sectional survey of current clinical practice. J Foot Ankle Res 13:14. 10.1186/s13047-020-0382-6 32164759 10.1186/s13047-020-0382-6 PMC7068881

[43] Rutten GMJ, Harting J, Rutten STJ, Bekkering GE, Kremers SPJ (2006) Measuring physiotherapists’ guideline adherence by means of clinical vignettes: a validation study. J Eval Clin Pract 12:491–500. 10.1111/j.1365-2753.2006.00699.x 16987111 10.1111/j.1365-2753.2006.00699.x

[44] Hughes R, Huby M (2002) The application of vignettes in social and nursing research. J Adv Nurs 37:382–386. 10.1046/j.1365-2648.2002.02100.x 11872108 10.1046/j.1365-2648.2002.02100.x

[45] NICE guidelines [NG226] (2022) Osteoarthritis in over 16s: diagnosis and management. https://www.nice.org.uk/guidance/ng226 Accessed 24 Nov 2024

[46] Guidelines for the non-surgical management of hip and knee osteoarthritis (2018) Royal Australian College of General Practitioners (RACGP). https://www.racgp.org.au/clinical-resources/clinical-guidelines/key-racgp-guidelines/view-all-racgp-guidelines/knee-and-hip-osteoarthritis Accessed 24 Nov 2024

[47] Keiserman LS, Cassandra J, Amis JA (2003) The piano key test: a clinical sign for the identification of subtle tarsometatarsal pathology. Foot Ankle Int 24:437–438. 10.1177/107110070302400511 12801202 10.1177/107110070302400511

[48] Molyneux P, Stewart S, Bowen C, Ellis R, Rome K, Carroll M (2022) A bibliometric analysis of published research employing musculoskeletal imaging modalities to evaluate foot osteoarthritis. J Foot Ankle Res 15:39. 10.1186/s13047-022-00549-0 35596206 10.1186/s13047-022-00549-0 PMC9121542

[49] Lithgow MJ, Buldt AK, Munteanu SE et al (2024) Plantar pressures in people with midfoot osteoarthritis: cross-sectional findings from the clinical assessment study of the foot. Gait Posture 108:243–249. 10.1016/j.gaitpost.2023.12.008 38141537 10.1016/j.gaitpost.2023.12.008

[50] Munteanu SE, Zammit GV, Menz HB (2012) Factors associated with foot pain severity and foot-related disability in individuals with first metatarsophalangeal joint OA. Rheumatology 51:176–183. 10.1093/rheumatology/ker344 22096012 10.1093/rheumatology/ker344

[51] Australian Government Department of Health and Aged Care: podiatrists 2019 factsheet (2024). https://hwd.health.gov.au/resources/publications/factsheet-alld-podiatrists-2019.pdf. Accessed 10 Dec 2024

