# Supplementary material for: Assessment and management of midfoot osteoarthritis by podiatrists in Australia: a cross-sectional survey of current practice
Source: Rheumatol Int. 2025 May 12;45(6):141. doi: 10.1007/s00296-025-05881-9 (PMC12069427; doi:10.1007/s00296-025-05881-9)
Supplement: Supplementary file 1 — Supplementary file1 (PDF 1092 KB) [file 296_2025_5881_MOESM1_ESM.pdf]

Management of midfoot osteoarthritis by podiatrists in Australia: a cross-sectional survey of current clinical practice

Thank you for considering to participate in this survey.

We are seeking the views of podiatrists who treat patients with symptomatic midfoot osteoarthritis (OA) and are interested in your clinical opinions on diagnosis, assessment and management of this condition. The survey will take approximately 15-20 minutes.

Please read the 'Participant Information and Consent Form' below. You can indicate that you 'agree to start the survey' at the end of this form.

Ethics reference number: HEC22329
Approval Period: until 21 November 2027

Participant Information and Consent Form

Management of midfoot osteoarthritis by podiatrists in Australia: a cross-sectional survey of current clinical practice.

This research is being carried out in partial fulfilment of a PhD by Ms Polly Lim under the supervision of Professor Shannon Munteanu. The following researchers will be conducting the study:

Role

Name

Organisation

Student investigator

Ms Polly Lim

Discipline of Podiatry, School of Allied Health, Human Services and Sport, La Trobe University

Principal investigator

Professor Shannon Munteanu

Discipline of Podiatry, School of Allied Health, Human Services and Sport, La Trobe University

Co-investigator

Professor Hylton Menz

Discipline of Podiatry, School of Allied Health, Human Services and Sport, La Trobe University

Co-investigator

Professor Karl Landorf

Discipline of Podiatry, School of Allied Health, Human Services and Sport, La Trobe University

Co-investigator

Dr Kade Paterson

Department of Physiotherapy, Melbourne School of Health Sciences, Centre for Health, Exercise and Sports Medicine, The University of Melbourne

Research funder

This study is funded by an Australian Government Research Training Program Scholarship 2021.

What is the study about?

You are invited to participate in an online survey to seek the views and experiences of podiatrists who treat people

with symptomatic midfoot osteoarthritis (OA). By symptomatic midfoot OA, we mean pain and associated symptoms of clinically diagnosed midfoot OA that may or may not have been confirmed by radiology. We hope to learn more about the usual care of people with midfoot OA - from a podiatrist's perspective. This is an anonymous survey that will not ask for any identifier information.

The aim of our study is to identify the common approaches to diagnosing, assessing, and managing symptoms associated with midfoot OA. Findings from this study will provide grounding for (i) the design of future clinical trials for this condition, and (ii) future conservative treatments and management guidelines for this condition.

Do I have to participate?

Being part of this study is voluntary. If you want to be part of the study, we ask that you read the information below carefully.

You can read the information below and decide at the end if you do not want to participate. If you decide not to participate, this will not affect your relationship with La Trobe University, or any other organisation listed.

Who is being asked to participate?

You have been asked to participate because you are a podiatrist. To be included in this study, you must:

(i) be a registered podiatrist based in Australia; and,

(ii) have managed at least one case of symptomatic midfoot OA in the last 6 months.

What will I be asked to do?

If you want to take part in this study, you will need to complete this online survey. It will take approximately 15 minutes of your time.

The first section of the online survey will include anonymous demographic questions on your age, gender, education, and clinical practice. The following four sections of the online survey will present a case vignette of a typical individual with midfoot OA and ask questions on how you would diagnose, assess, and manage this individual.

It is advised to complete the online survey on a bigger device such as a desktop or laptop, so that you can easily refer to the case vignette when answering the questions.

What are the benefits?

The benefit of you taking part in this study is that you will be able to contribute your views and approach to diagnosing, assessing, and managing midfoot OA. Key findings from this study will guide the design of research trials that reflect current clinical practice. The findings of these trials will be useful to develop clinical guidelines that are of relevance to you and other healthcare professionals.

What are the risks?

With any study there are (1) risks we know about, (2) risks we don't know about and (3) risks we don't expect. If you experience something that you aren't sure about, please contact us immediately so we can discuss the best way to manage your concerns.

Name/Organisation

Position

Telephone

Email

Ms Polly Lim, La Trobe University

Professor Shannon Munteanu, La Trobe University

Student investigator,

PhD Candidate

Principal investigator,

Primary supervisor

+61 3 9479 1411

+61 3 9479 5866

p.lim@latrobe.edu.au

s.munteanu@latrobe.edu.au

We do not foresee any risks associated with this study.

What will happen to information about me?

By selecting 'Yes' to 'I agree to start the survey' at the end of this form, this tells us that you want to take part in the study.

We will collect information about you in ways that will not reveal who you are.

We will store information about you in ways that will not reveal who you are.

We will publish information about you in ways that will not be identified in any type of publication from this study.

We will keep your information indefinitely in an open access repository.

The storage, transfer and destruction of your data will be undertaken in accordance with the La Trobe Research Data Management Policy https://policies.latrobe.edu.au/document/view.php?id=106.

Any personal information you provide will be handled in accordance with applicable privacy laws, any health information collected will be handled in accordance with the Health Records Act 2001 (Vic). Subject to any exceptions in relevant laws, you have the right to access and correct your personal information by contacting the research team.

Will I hear about the results of the study?

You will not be contacted directly about the results as we will not be collecting any identifier information from you. Findings from the survey will be disseminated as broadly as possible to allow access by you, other healthcare professionals, researchers, and the wider community. This will be achieved by: (i) publication of resulting manuscripts in open-access journals; (ii) use of social media; (iii) presentations at relevant conferences; (iv) sharing

of publications through the associations and groups that we advertised the survey through and; (v) implementing the findings into undergraduate, postgraduate and professional development education programs for relevant health professionals around Australia.

What if I change my mind?

If you no longer want to complete the survey, simply close the web browser at any point in time. We cannot withdraw your responses once you've fully submitted the survey because we will be unable to link who you are with your survey responses.

Your decision to leave the survey or withdraw at any point will not affect your relationship with the investigators conducting the study, La Trobe University, or any other organisation listed.

Who can I contact for questions or want more information?

If you would like to speak to us, please use the contact details below:

Name/Organisation

Position

Telephone

Email

Ms Polly Lim, La Trobe University

Professor Shannon Munteanu, La Trobe University

Student investigator,

PhD Candidate

Principal investigator,

Primary supervisor

+61 3 9479 1411

+61 3 9479 5866

p.lim@latrobe.edu.au

s.munteanu@latrobe.edu.au

What if I have a complaint?

If you have a complaint about any part of this study, please contact:

Ethics Reference Number

Position

Telephone

Email

HEC22329

Senior Research Ethics Officer

+61 3 9479 1443

humanethics@latrobe.edu.au

Consent Form - Declaration by Participant

I (the participant) have read and understood the Participant Information Statement, and any questions have been answered to my satisfaction. I agree to participate in the study, I know I can withdraw at any time until completion of the online survey. I agree information provided by me or with my permission during the project may be included in a thesis, presentation and published in journals on the condition that I cannot be identified.

◯ I would like my information collected for this research study to be used in any future studies.

Have you completed this survey before?	◯ Yes<br>◯ No

I agree to start the survey:

◯ Yes
◯ No

Are you currently an AHPRA registered podiatrist practising in Australia?	◯ Yes<br>◯ No
We are seeking the views of podiatrists who treat people with symptomatic midfoot osteoarthritis (OA) in Australia.<br><br>Have you managed at least one case of symptomatic midfoot OA in the last 6 months?	◯ Yes<br>◯ No

---

Thank you for your interest in our research. Unfortunately, you do not meet the eligibility criteria for this survey.

Section One: About you

Which state do you practise most in?	◯ ACT<br>◯ NSW<br>◯ NT<br>◯ QLD<br>◯ SA<br>◯ TAS<br>◯ VIC<br>◯ WA

Please select from the range (in years) when you received your podiatry qualifications.	◯ 2021 to present<br>◯ 2016 to 2020<br>◯ 2011 to 2015<br>◯ 2006 to 2010<br>◯ 2001 to 2005<br>◯ 1996 to 2000<br>◯ 1991 to 1995<br>◯ 1985 to 1990<br>◯ 1981 to 1985<br>◯ 1976 to 1980<br>◯ 1971 to 1975<br>◯ 1966 to 1970<br>◯ 1961 to 1965
Which educational institution did you obtain your initial podiatry qualifications from?	◯ Central Queensland University<br>◯ Charles Sturt University<br>◯ La Trobe University (previously Lincoln Institute of Health Sciences)<br>◯ University of Newcastle Australia<br>◯ University of Western Australia (previously Curtin University of Technology)<br>◯ Queensland University of Technology<br>◯ Southern Cross University<br>◯ University of South Australia (previously South Australian Institute of Technology)<br>◯ Western Sydney University (previously University of Western Sydney)<br>◯ Other
You selected 'Other', please specify the name of your educational institution:	__________________________________
What is your gender?	◯ Man<br>◯ Woman<br>◯ Non-binary<br>◯ Prefer to self-describe: ______<br>◯ I prefer not to answer

What is your current age?

- ◯ 21
- ◯ 22
- ◯ 23
- ◯ 24
- ◯ 25
- ◯ 26
- ◯ 27
- ◯ 28
- ◯ 29
- ◯ 30
- ◯ 31
- ◯ 32
- ◯ 33
- ◯ 34
- ◯ 35
- ◯ 36
- ◯ 37
- ◯ 38
- ◯ 39
- ◯ 40
- ◯ 41
- ◯ 42
- ◯ 43
- ◯ 44
- ◯ 45
- ◯ 46
- ◯ 47
- ◯ 48
- ◯ 49
- ◯ 50
- ◯ 51
- ◯ 52
- ◯ 53
- ◯ 54
- ◯ 55
- ◯ 56
- ◯ 57
- ◯ 58
- ◯ 59
- ◯ 60
- ◯ 61
- ◯ 62
- ◯ 63
- ◯ 64
- ◯ 65
- ◯ 66
- ◯ 67
- ◯ 68
- ◯ 69
- ◯ 70
- ◯ 71
- ◯ 72
- ◯ 73
- ◯ 74
- ◯ 75
- ◯ 76
- ◯ 77
- ◯ 78
- ◯ 79
- ◯ 80
- ◯ 81
- ◯ 82
- ◯ 83
- ◯ 84
- ◯ 85
- ◯ 86
- ◯ 87
- ◯ 88
- ◯ 89

	◯ 90
Do you currently work...	◯ Exclusively in public health system<br>◯ Exclusively in private health settings<br>◯ In both public and non-public settings
How many clinical equivalent full-time (EFT) years have you worked in total? E.g. 1 EFT = 1 year, 0.5 EFT for two years = 1 year; 0.2 EFT for five years = 1 year.	◯ Less than 1 year<br>◯ 1<br>◯ 2<br>◯ 3<br>◯ 4<br>◯ 5<br>◯ 6<br>◯ 7<br>◯ 8<br>◯ 9<br>◯ 10<br>◯ 11<br>◯ 12<br>◯ 13<br>◯ 14<br>◯ 15<br>◯ 16<br>◯ 17<br>◯ 18<br>◯ 19<br>◯ 20<br>◯ 21<br>◯ 22<br>◯ 23<br>◯ 24<br>◯ 25<br>◯ 26<br>◯ 27<br>◯ 28<br>◯ 29<br>◯ 30<br>◯ 31<br>◯ 32<br>◯ 33<br>◯ 34<br>◯ 35<br>◯ 36<br>◯ 37<br>◯ 38<br>◯ 39<br>◯ 40<br>◯ 41<br>◯ Greater than 41 years
Last week, what was your principal role in your main job in Podiatry?	◯ Clinician (including managers and supervisors also providing clinical services)<br>◯ Administrator (including managers not providing clinical services)<br>◯ Teacher or educator<br>◯ Researcher<br>◯ Other, please specify: ______

On average, during the past 6 months, how many hours (per week) do you spend working in a clinical environment?	◯ Less than 1 hour per week<br>◯ 1<br>◯ 2<br>◯ 3<br>◯ 4<br>◯ 5<br>◯ 6<br>◯ 7<br>◯ 8<br>◯ 9<br>◯ 10<br>◯ 11<br>◯ 12<br>◯ 13<br>◯ 14<br>◯ 15<br>◯ 16<br>◯ 17<br>◯ 18<br>◯ 19<br>◯ 20<br>◯ 21<br>◯ 22<br>◯ 23<br>◯ 24<br>◯ 25<br>◯ 26<br>◯ 27<br>◯ 28<br>◯ 29<br>◯ 30<br>◯ 31<br>◯ 32<br>◯ 33<br>◯ 34<br>◯ 35<br>◯ 36<br>◯ 37<br>◯ 38<br>◯ 39<br>◯ 40<br>◯ 41<br>◯ 42<br>◯ 43<br>◯ 44<br>◯ Greater than 44 hours per week
Over the past 6 months, what percentage of your caseload would you estimate to be managing musculoskeletal conditions of the foot and/or ankle and/or lower leg?	0% 100%<br>*(Place a mark on the scale above)*
Over the past 6 months, how frequently do you manage people with symptomatic midfoot osteoarthritis?	◯ at most 1 in the past 6 months<br>◯ at most 2-3 in the past 6 months<br>◯ at most 4-6 in the past 6 months<br>◯ at most 7-12 in the past 6 months<br>◯ at most 13-24 in the past 6 months<br>◯ greater than 24 in the past 6 months
Have you received any postgraduate training that might be relevant for managing osteoarthritis?	◯ Yes<br>◯ No

If yes, please select all that apply.	☐ Day or weekend courses with no formal assessment<br>☐ Non-university courses or modules with formal assessments (exam, marked assignments etc.)<br>☐ University post graduate (postgraduate certificate, diploma, master, doctorate), or equivalent<br>☐ Other(s), please specify: ______
Are you a podiatrist who is endorsed for prescribing scheduled medicines?	◯ Yes<br>◯ No
If yes, which year did you obtain your endorsement?	◯ 2023<br>◯ 2022<br>◯ 2021<br>◯ 2020<br>◯ 2019<br>◯ 2018<br>◯ 2017<br>◯ 2016<br>◯ 2015<br>◯ 2014<br>◯ 2013<br>◯ 2012<br>◯ 2011<br>◯ 2010

Assessment: Diagnosis

Clinical scenario of a patient with symptomatic midfoot osteoarthritis (OA).

Presented below is a clinical scenario of a patient with midfoot OA pain, who you see for the first time. All questions that follow relate to the assessment and care you would give this particular patient. Think about the entire course of the patient's typical consultation(s) with you when referring to the case vignette as described below.

A 63-year-old woman was referred from her general practitioner due to right dorsum midfoot pain which has worsened over the past 6 months. Her general practitioner diagnosed her as having midfoot osteoarthritis.

She takes pain relief medications occasionally and has not had any previous treatment for her foot pain. She is mildly overweight, has arthritis in her right shoulder and takes daily medication for hyperlipidaemia and hypertension. She is a retired production line factory worker and typically spends 4 hours per day engaging in light physical activities including gardening. More recently, she has been unable to participate in her routine daily activities. She rates the severity of her pain at the midfoot as 6 out of 10 at worst and states that the pain is aggravated by walking and climbing stairs.

Examination findings include, a moderately pronated foot posture of both feet and some pain with passive movement of the midfoot articulations. On gait analysis, it is observed that she has a wide base of gait and limps due to her foot pain.

Section 2: Diagnosis

In this section, we will ask questions relating to the diagnosis of midfoot OA. All questions that follow relate to the assessment and care you would provide to the person as described in the clinical scenario above.

How do you diagnose midfoot OA? (Select all that apply)	☐ Based on the healthcare professional's referral<br>☐ Physical assessment (history taking and objective assessment)<br>☐ Imaging<br>☐ Other(s), please specify: ______
Do you conduct any patient reported outcome measures (PROMs)? e.g. numerical rating scale (NRS), visual analogue scale (VAS), Foot Health Status Questionnaire (FHSQ), Manchester Foot Pain and Disability Index (MFPDI) etc.	◯ Yes<br>◯ No
If yes, please select the PROM(s) used:	☐ Numerical rating scale (NRS)<br>☐ Visual analogue scale (VAS)<br>☐ Foot Health Status Questionnaire (FHSQ)<br>☐ Manchester Foot Pain and Disability Index (MFPDI)<br>☐ Other(s), please specify: ______

Right Foot

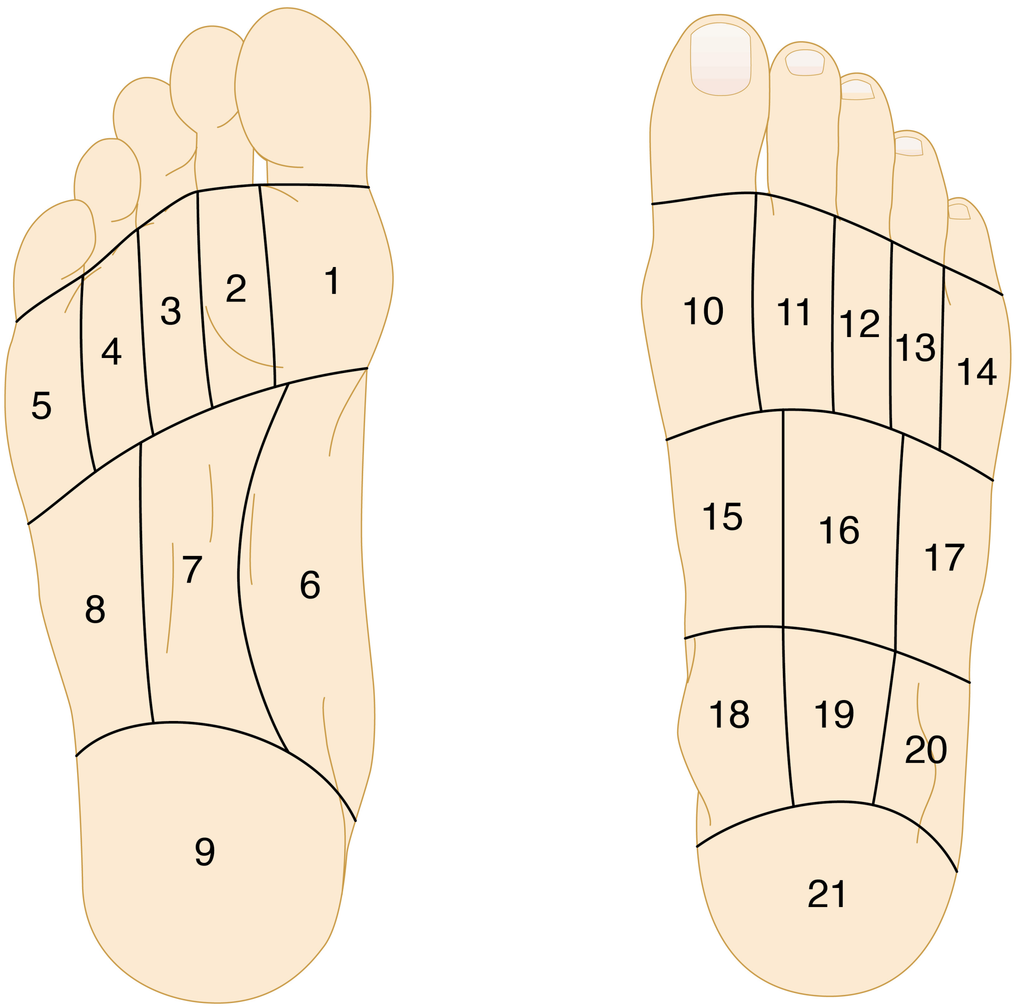

Plantar

Dorsal

Referring to the foot diagram (Right foot) above, please select the corresponding number(s) you would typically consider represents the location of midfoot OA pain? (Please select all that apply)	☐ 1<br>☐ 2<br>☐ 3<br>☐ 4<br>☐ 5<br>☐ 6<br>☐ 7<br>☐ 8<br>☐ 9<br>☐ 10<br>☐ 11<br>☐ 12<br>☐ 13<br>☐ 14<br>☐ 15<br>☐ 16<br>☐ 17<br>☐ 18<br>☐ 19<br>☐ 20<br>☐ 21
What components of a musculoskeletal exam do you typically use as part of your diagnostic process for individuals with midfoot OA (like this woman presented in this case)? (Please select all that apply)	☐ Visual inspection<br>☐ Passive movement testing<br>☐ Resisted isometric muscle testing<br>☐ Active movement testing<br>☐ Palpation<br>☐ Other(s), please specify: ______
Do you typically use imaging as part of your diagnostic procedure?	◯ Yes<br>◯ No
If no, please provide reason(s) why:	__________________________________
What imaging modality(ies) will you typically use? (Select all that apply)	☐ X-ray<br>☐ Ultrasound<br>☐ MRI<br>☐ CT<br>☐ Bone scan<br>☐ Other, please specify: ______
Do you use any real-time imaging e.g. ultrasound in clinic?	◯ Yes<br>◯ No
Which imaging modality will you typically want to perform first?	◯ X-ray<br>◯ Ultrasound<br>◯ MRI<br>◯ CT<br>◯ Bone scan<br>◯ [imaging_other]
Do you typically refer individuals with midfoot OA (like this woman presented in the case) to a medical practitioner (e.g. GP or specialist) for further investigations to assist diagnosis?	◯ Yes<br>◯ No
Who will you typically refer to for further investigations? (State the profession)	__________________________________

What further investigations would you typically refer her for? (State the type of investigation)	__________________________________
Are there any special test(s) you would perform to confirm the diagnosis of midfoot OA?	◯ Yes, please specify: ______<br>◯ No

Assessment: Associated impairments and comorbidities

Clinical scenario of a patient with symptomatic midfoot osteoarthritis (OA).

Presented below is the same patient with midfoot OA pain, who you see for the first time. All questions that follow relate to the assessment and care you would give this particular patient. Think about the entire course of the patient's typical consultation(s) with you when referring to the case vignette as described below.

A 63-year-old woman was referred from her general practitioner due to right dorsum midfoot pain which has worsened over the past 6 months. Her general practitioner diagnosed her as having midfoot osteoarthritis.

She takes pain relief medications occasionally and has not had any previous treatment for her foot pain. She is mildly overweight, has arthritis in her right shoulder and takes daily medication for hyperlipidaemia and hypertension. She is a retired production line factory worker and typically spends 4 hours per day engaging in light physical activities including gardening. More recently, she has been unable to participate in her routine daily activities. She rates the severity of her pain at the midfoot as 6 out of 10 at worst and states that the pain is aggravated by walking and climbing stairs.

Examination findings include, a moderately pronated foot posture of both feet and some pain with passive movement of the midfoot articulations. On gait analysis, it is observed that she has a wide base of gait and limps due to her foot pain.

Section 3: Assessment

In this section, we will ask questions relating to associated factors, physical impairments and/or comorbidities you would look out for when assessing this patient. All questions that follow relate to the assessment and care you would provide to the same person as described in the clinical scenario above.

What tests do you typically perform to assess for physical impairments (to identify associated factors)? (Please select all that apply).	☐ Range of motion<br>☐ Static alignment<br>☐ Muscle strength<br>☐ Gait analysis<br>☐ Functional tests<br>☐ Other
You have selected 'Other'. Please specify other assessments you would typically perform to assess for physical impairments:	__________________________________
You have selected 'Range of motion'. Please select all that apply.	☐ Ankle<br>☐ Midfoot<br>☐ 1st metatarsophalangeal joint<br>☐ Other(s), please specify: ______
You have selected 'Static alignment'. Please select all that apply.	☐ Foot posture<br>☐ Other(s), please specify: ______
You have selected 'Muscle strength'. Please select all that apply.	☐ Ankle plantarflexors<br>☐ Ankle dorsiflexors<br>☐ Ankle invertors<br>☐ Ankle evertors<br>☐ Lesser toes plantarflexors<br>☐ Hallux plantarflexors<br>☐ Other(s), please specify: ______

You have selected 'Gait analysis'. Please select all that apply.	☐ Visual<br>☐ Computerised kinematics<br>☐ Computerised kinetics<br>☐ Plantar pressures<br>☐ Other(s), please specify: ______
You have selected 'Functional tests'. Please select all that apply.	☐ Calf raises<br>☐ Hops<br>☐ Stairs<br>☐ Balance test<br>☐ Other(s), please specify: ______
Do you typically assess this patient's body composition (e.g. weight, BMI) as part of your consult?	◯ Yes<br>◯ No
If yes, how would you do it? Please select all that apply.	☐ Observation<br>☐ Measure waist/ hip circumference<br>☐ Ask the patient to tell you her weight<br>☐ Calculate Body Mass Index<br>☐ Measure the patient's weight using scales<br>☐ Other(s), please specify: ______
Do you typically assess this patient for the presence of comorbidities?	◯ Yes<br>◯ No
Do you typically assess for cognitive / psychological factors?	◯ Yes<br>◯ No

Non-surgical treatment approaches

Clinical scenario of a patient with symptomatic midfoot osteoarthritis (OA).

Presented below is the same patient with midfoot OA pain, who you see for the first time. All questions that follow relate to the care you would give this particular patient. Think about the entire course of the patient's typical consultation(s) with you when referring to the case vignette as described below.

A 63-year-old woman was referred from her general practitioner due to right dorsum midfoot pain which has worsened over the past 6 months. Her general practitioner diagnosed her as having midfoot osteoarthritis.

She takes pain relief medications occasionally and has not had any previous treatment for her foot pain. She is mildly overweight, has arthritis in her right shoulder and takes daily medication for hyperlipidaemia and hypertension. She is a retired production line factory worker and typically spends 4 hours per day engaging in light physical activities including gardening. More recently, she has been unable to participate in her routine daily activities. She rates the severity of her pain at the midfoot as 6 out of 10 at worst and states that the pain is aggravated by walking and climbing stairs.

Examination findings include, a moderately pronated foot posture of both feet and some pain with passive movement of the midfoot articulations. On gait analysis, it is observed that she has a wide base of gait and limps due to her foot pain.

Section 4: Non-surgical management

We are interested in your most common practices concerning non-surgical approaches for the treatment of symptomatic midfoot OA. When completing the following questions, the patient is the same person as described in the clinical scenario above.

In the initial 6 weeks, what non-surgical approaches will you usually use to treat this patient? Tick all the boxes that reflect the treatment you will typically aim to provide to this patient.

- ☐ Cryotherapy (Cold therapy)
- ☐ Dry needling
- ☐ Education
- ☐ Extracorporeal shock wave therapy (ESWT/FSWT/RSWT)
- ☐ Exercise therapy (including advice, strengthening, range of motion, aerobic exercise)
- ☐ Foot mobilisation and manipulation therapy
- ☐ Footwear advice
- ☐ Gait re-training
- ☐ Laser therapy
- ☐ Massage
- ☐ Orthotic therapy
- ☐ Padding
- ☐ Pharmacological therapy (including topical and oral analgesics, injection therapy and, complementary therapies and nutraceuticals)
- ☐ Strapping / Taping
- ☐ Thermotherapy (Heat therapy)
- ☐ Trigger point techniques
- ☐ Other(s)

You selected 'Other(s)', please list a non-surgical treatment modality (that is not listed above) you will typically aim to provide in the initial 6 weeks. Please select/tick and detail one modality per box.

You are not required to fill in every box.

- ☐ Other(1), please specify: ______
- ☐ Other(2), please specify: ______
- ☐ Other(3), please specify: ______
- ☐ Other(4), please specify: ______
- ☐ Other(5), please specify: ______

From the above, rank your top 5 treatment modalities you would typically aim to provide this patient initially (i.e. first 6 weeks). You can select 'Nil' if you have less than five rankings.

Top 5 (Rank 1)

- ◯ Cryotherapy (Cold therapy)
- ◯ Dry needling
- ◯ Education
- ◯ Extracorporeal shock wave therapy (ESWT/FSWT/RSWT)
- ◯ Exercise therapy (including advice, strengthening, range of motion, aerobic exercise)
- ◯ Foot mobilisation and manipulation therapy
- ◯ Footwear advice
- ◯ Gait re-training
- ◯ Laser therapy
- ◯ Massage
- ◯ Orthotic therapy
- ◯ Padding
- ◯ Pharmacological therapy (including topical and oral analgesics, injection therapy and, complementary therapies and nutraceuticals)
- ◯ Strapping / Taping
- ◯ Thermotherapy (Heat therapy)
- ◯ Trigger point techniques
- ◯ [treatment_other1]
- ◯ [treatment_other2]
- ◯ [treatment_other3]
- ◯ [treatment_other4]
- ◯ [treatment_other5]

Top 5 (Rank 2)

- ◯ Cryotherapy (Cold therapy)
- ◯ Dry needling
- ◯ Education
- ◯ Extracorporeal shock wave therapy (ESWT/FSWT/RSWT)
- ◯ Exercise therapy (including advice, strengthening, range of motion, aerobic exercise)
- ◯ Foot mobilisation and manipulation therapy
- ◯ Footwear advice
- ◯ Gait re-training
- ◯ Laser therapy
- ◯ Massage
- ◯ Orthotic therapy
- ◯ Padding
- ◯ Pharmacological therapy (including topical and oral analgesics, injection therapy and, complementary therapies and nutraceuticals)
- ◯ Strapping / Taping
- ◯ Thermotherapy (Heat therapy)
- ◯ Trigger point techniques
- ◯ [treatment_other1]
- ◯ [treatment_other2]
- ◯ [treatment_other3]
- ◯ [treatment_other4]
- ◯ [treatment_other5]
- ◯ Nil

Top 5 (Rank 3)	◯ Cryotherapy (Cold therapy)<br>◯ Dry needling<br>◯ Education<br>◯ Extracorporeal shock wave therapy (ESWT/FSWT/RSWT)<br>◯ Exercise therapy (including advice, strengthening, range of motion, aerobic exercise)<br>◯ Foot mobilisation and manipulation therapy<br>◯ Footwear advice<br>◯ Gait re-training<br>◯ Laser therapy<br>◯ Massage<br>◯ Orthotic therapy<br>◯ Padding<br>◯ Pharmacological therapy (including topical and oral analgesics, injection therapy and, complementary therapies and nutraceuticals)<br>◯ Strapping / Taping<br>◯ Thermotherapy (Heat therapy)<br>◯ Trigger point techniques<br>◯ [treatment_other1]<br>◯ [treatment_other2]<br>◯ [treatment_other3]<br>◯ [treatment_other4]<br>◯ [treatment_other5]<br>◯ Nil
Top 5 (Rank 4)	◯ Cryotherapy (Cold therapy)<br>◯ Dry needling<br>◯ Education<br>◯ Extracorporeal shock wave therapy (ESWT/FSWT/RSWT)<br>◯ Exercise therapy (including advice, strengthening, range of motion, aerobic exercise)<br>◯ Foot mobilisation and manipulation therapy<br>◯ Footwear advice<br>◯ Gait re-training<br>◯ Laser therapy<br>◯ Massage<br>◯ Orthotic therapy<br>◯ Padding<br>◯ Pharmacological therapy (including topical and oral analgesics, injection therapy and, complementary therapies and nutraceuticals)<br>◯ Strapping / Taping<br>◯ Thermotherapy (Heat therapy)<br>◯ Trigger point techniques<br>◯ [treatment_other1]<br>◯ [treatment_other2]<br>◯ [treatment_other3]<br>◯ [treatment_other4]<br>◯ [treatment_other5]<br>◯ Nil

Top 5 (Rank 5)	◯ Cryotherapy (Cold therapy)<br>◯ Dry needling<br>◯ Education<br>◯ Extracorporeal shock wave therapy (ESWT/FSWT/RSWT)<br>◯ Exercise therapy (including advice, strengthening, range of motion, aerobic exercise)<br>◯ Foot mobilisation and manipulation therapy<br>◯ Footwear advice<br>◯ Gait re-training<br>◯ Laser therapy<br>◯ Massage<br>◯ Orthotic therapy<br>◯ Padding<br>◯ Pharmacological therapy (including topical and oral analgesics, injection therapy and, complementary therapies and nutraceuticals)<br>◯ Strapping / Taping<br>◯ Thermotherapy (Heat therapy)<br>◯ Trigger point techniques<br>◯ [treatment_other1]<br>◯ [treatment_other2]<br>◯ [treatment_other3]<br>◯ [treatment_other4]<br>◯ [treatment_other5]<br>◯ Nil
You selected 'Exercise therapy' as part of your treatment approach.<br><br>Select the options that reflect what exercises / advice you will typically provide. (Please select all that apply).	☐ Foot and ankle extrinsic muscle strengthening<br>☐ Intrinsic foot muscle strengthening<br>☐ Modification of physical activity<br>☐ Proprioception/balance<br>☐ Self mobilisation<br>☐ Self myofascial release (e.g. foam rolling)<br>☐ Stretching<br>☐ Refer to others for exercise therapy<br>☐ Other(s), please specify: ______
You selected 'Modification of physical activity'. (Please select all that apply).	☐ Alternative exercises e.g. General advice around low impact exercise such as swimming<br>☐ Avoidance of aggravating activity<br>☐ Increasing activity<br>☐ Pacing of activities (to do a little bit often)<br>☐ Reducing activity<br>☐ Other(s), please specify: ______
You selected 'Refer to others for exercise therapy'. Specify who (i.e. profession) you would typically refer to.	______________________________
You selected 'Refer to others for exercise therapy'. Specify what (i.e. type of exercise) you would typically refer for?	______________________________

You selected 'Footwear advice'. (Please select all that apply).	☐ Adequate length<br>☐ Adequate width<br>☐ Adequate depth<br>☐ Adequate fixation<br>☐ Rigid heel counter<br>☐ Rigid midsole<br>☐ Rocker bottom outsole<br>☐ Minimalist 'barefoot'<br>☐ Cushioned<br>☐ Other(s), please specify: ______
You selected 'Orthotic therapy'. (Please select all that apply).	☐ Prefabricated<br>☐ Custom-made
Please select any 'Prefabricated' foot orthoses that you will typically prescribe. (Please select all that apply).	☐ Arch contouring<br>☐ Shoe stiffening (full length)<br>☐ Other(s), please specify: ______
Do you modify the prefabricated device?	◯ Yes<br>◯ No
What modifications do you apply?	__________________________________
You selected 'Padding', please specify what techinique/type of padding:	__________________________________
Please select any 'Custom-made' foot orthoses that you will typically prescribe. (Please select all that apply).	☐ Arch contouring<br>☐ Shoe stiffening (full length)<br>☐ Other(s), please specify: ______
List 1 device you would typically prescribe or recommend first.	◯ Prefabricated arch contouring foot orthoses<br>◯ Prefabricated shoe stiffening inserts<br>◯ [prefab_other]<br>◯ Custom-made arch contouring foot orthoses<br>◯ Custom-made shoe stiffening inserts<br>◯ [custom_other]
You selected 'Strapping / Taping', what technique do you apply. (Please select all that apply).	☐ Low dye<br>☐ High dye<br>☐ J strap<br>☐ Other(s), please specify: ______
What type of taping (strapping) would your typically use? (Please select all that apply).	☐ Rigid tape<br>☐ Kinesiology tape<br>☐ Other(s), please specify: ______
You selected 'Pharmacological therapy'. What pharmacological approach do you typically use or advise? (Please select all that apply).	☐ Topical therapies<br>☐ Oral analgesics<br>☐ Injection therapy<br>☐ Complementary therapies and nutraceuticals
You selected 'Topical therapies'. (Please select all that apply).	☐ Non-steroidal anti-inflammatory drugs (NSAIDs)<br>☐ Capsaicin<br>☐ Other(s), please specify: ______

You selected 'Oral analgesics'. (Please select all that apply).	☐ Paracetamol<br>☐ Non-steroidal anti-inflammatory drugs (NSAIDs)<br>☐ Other(s), please specify: ______
You selected 'Injection therapy'. What pharmacological agent would you use or primarily recommend for the injection therapy?	◯ Corticosteroid<br>◯ Hyaluronan<br>◯ Other(s), please specify: ______
Will you typically perform the injection therapy yourself?	◯ Yes<br>◯ No
Who will you typically refer the patient to for the injection therapy? Select all that apply.<br><br>Select N/A if you'd perform the injection yourself.	☐ Foot and ankle orthopaedic surgeon<br>☐ GP<br>☐ Podiatric surgeon<br>☐ Radiologist<br>☐ Sports physician<br>☐ Other(s), please specify: ______<br>☐ N/A
You selected 'Complementary therapies and nutraceuticals'. (Please select all that apply).	☐ Glucosamine<br>☐ Chondroitin<br>☐ Tumeric/curcumin<br>☐ Other(s), please specify: ______
Do you typically address this patient's body composition (e.g. weight, BMI) as part of your treatment?	◯ Yes<br>◯ No
If yes, please select all that apply.	☐ Provide advice about weight loss<br>☐ Actively deliver a weight loss program<br>☐ Refer on to a dietitian<br>☐ Refer on to a weight loss program<br>☐ Other(s) please specify: ______
Team approach<br><br>Will you likely refer to other healthcare professional(s) to co-manage this patient in the initial 6 weeks?	◯ Yes<br>◯ No
If yes, select the boxes that reflect your referral practices. (Please select all that apply).	☐ Acupuncturist<br>☐ Chiropractor<br>☐ Dietitian<br>☐ Exercise physiologist<br>☐ GP<br>☐ Myotherapist<br>☐ Occupational therapist<br>☐ Other Podiatrist(s)<br>☐ Orthopaedic surgeon<br>☐ Osteopath<br>☐ Pain clinic<br>☐ Pharmacist<br>☐ Physiotherapist<br>☐ Podiatric surgeon<br>☐ Rheumatologist<br>☐ Sports physician<br>☐ Other(s), please specify: ______

Treatment timeline

Clinical scenario of a patient with symptomatic midfoot osteoarthritis (OA).

Presented below is the same patient with midfoot OA pain, who you see for the first time. All questions that follow relate to the case vignette as described below.

A 63-year-old woman was referred from her general practitioner due to right dorsum midfoot pain which has worsened over the past 6 months. Her general practitioner diagnosed her as having midfoot osteoarthritis.

She takes pain relief medications occasionally and has not had any previous treatment for her foot pain. She is mildly overweight, has arthritis in her right shoulder and takes daily medication for hyperlipidaemia and hypertension. She is a retired production line factory worker and typically spends 4 hours per day engaging in light physical activities including gardening. More recently, she has been unable to participate in her routine daily activities. She rates the severity of her pain at the midfoot as 6 out of 10 at worst and states that the pain is aggravated by walking and climbing stairs.

Examination findings include, a moderately pronated foot posture of both feet and some pain with passive movement of the midfoot articulations. On gait analysis, it is observed that she has a wide base of gait and limps due to her foot pain.

Section 5: Treatment timeline

We are interested to know your treatment expectations for the management of symptomatic midfoot OA. When completing the following questions, the patient is the same person as described in the clinical scenario above.

How many times will you typically see this patient including both assessment and treatment for their midfoot pain?	◯ Once<br>◯ Twice<br>◯ 3-4 times<br>◯ 5-8 times<br>◯ More than 8 times
Over how long (in weeks) will you typically see this patient for their midfoot pain?	◯ 1-2 weeks<br>◯ 3-4 weeks<br>◯ 5-6 weeks<br>◯ 7-8 weeks<br>◯ 9-10 weeks<br>◯ More than 10 weeks
What will be a reasonable time frame that you would expect this patient to first achieve a satisfactory outcome (improvement in symptoms) with the treatment you would have typically provided?	◯ Within the first week<br>◯ Between 1 week and 1 month<br>◯ Between 1 and 2 months<br>◯ Between 2 and 3 months<br>◯ Between 3 and 6 months<br>◯ Between 6 and 12 months<br>◯ Greater than 12 months
If your treatment approach was not able to reduce the symptoms of midfoot OA to a satisfactory level, at what time point following your initial treatment will you consider referring this patient to another healthcare practitioner?	◯ Within the first week<br>◯ Between 1 week and 1 month<br>◯ Between 1 and 2 months<br>◯ Between 2 and 3 months<br>◯ Between 3 and 6 months<br>◯ Between 6 and 12 months<br>◯ Greater than 12 months<br>◯ Never

At the selected timepoint above, who would you consider referring this patient to? (Please select all that apply).	☐ Acupuncturist<br>☐ Chiropractor<br>☐ Dietician<br>☐ Exercise physiologist<br>☐ GP<br>☐ Myotherapist<br>☐ Occupational therapist<br>☐ Other Podiatrist(s)<br>☐ Orthopaedic surgeon<br>☐ Osteopath<br>☐ Pain clinic<br>☐ Pharmacist<br>☐ Physiotherapist<br>☐ Podiatric surgeon<br>☐ Rheumatologist<br>☐ Sports physician<br>☐ Other
You selected 'Other', please specify the profession:	______________________
From the above, rank your top 3 preferences you'd commonly refer to.	
First preference (Rank 1)	○ Acupuncturist<br>○ Chiropractor<br>○ Dietician<br>○ Exercise physiologist<br>○ GP<br>○ Myotherapist<br>○ Occupational therapist<br>○ Other Podiatrist(s)<br>○ Orthopaedic surgeon<br>○ Osteopath<br>○ Pain clinic<br>○ Pharmacist<br>○ Physiotherapist<br>○ Podiatric surgeon<br>○ Rheumatologist<br>○ Sports physician<br>○ [refer_other1]
Second preference (Rank 2)	○ Acupuncturist<br>○ Chiropractor<br>○ Dietician<br>○ Exercise physiologist<br>○ GP<br>○ Myotherapist<br>○ Occupational therapist<br>○ Other Podiatrist(s)<br>○ Orthopaedic surgeon<br>○ Osteopath<br>○ Pain clinic<br>○ Pharmacist<br>○ Physiotherapist<br>○ Podiatric surgeon<br>○ Rheumatologist<br>○ Sports physician<br>○ [refer_other1]<br>○ Nil

Third preference (Rank 3)	◯ Acupuncturist<br>◯ Chiropractor<br>◯ Dietician<br>◯ Exercise physiologist<br>◯ GP<br>◯ Myotherapist<br>◯ Occupational therapist<br>◯ Other Podiatrist(s)<br>◯ Orthopaedic surgeon<br>◯ Osteopath<br>◯ Pain clinic<br>◯ Pharmacist<br>◯ Physiotherapist<br>◯ Podiatric surgeon<br>◯ Rheumatologist<br>◯ Sports physician<br>◯ [refer_other1]<br>◯ Nil

Please click the 'SUBMIT' button to complete the survey.
