# Supplementary material for: Assessment and management of midfoot osteoarthritis by podiatrists in Australia: a cross-sectional survey of current practice
Source: Rheumatol Int. 2025 May 12;45(6):141. doi: 10.1007/s00296-025-05881-9 (PMC12069427; doi:10.1007/s00296-025-05881-9)
Supplement: Supplementary file 3 — Supplementary file3 (DOCX 22 KB) [file 296_2025_5881_MOESM3_ESM.docx]

**Table 2. Items for reporting survey studies [1].**

| **No.** | **Items** | **Notes** | **Page** |
| --- | --- | --- | --- |
| 1 | Title | • Reflect on the survey subject, target respondents (e.g., patients, specialists, public representatives), obtained results, and study design (online, non-web-based, cross-sectional, longitudinal). | 1 |
| 2 | Abstract | • Provide a structured abstract with an introduction, aims, results, and conclusion. | 1 |
| 3 | Keywords | • Add the term "surveys and questionnaires" along with subject keywords to increase retrieval of the survey report. | 1 |
| 4 | Introduction | • Analyze available evidence, relevant reviews, and surveys to justify the need for current study and questionnaire sections. | 2 |
| 5 | Aim | • Present specific and innovative aims. | 2 |
| 6 | Methods | • Highlight study design (e.g., web-based, non-web-based, cross-sectional, longitudinal). | 2 |
|  |  | • Specify the survey datelines and characterize time periods (data collection during a crisis [pandemic, wartime] or certain global movements, campaigns, or interventions). | 2 |
|  |  | • Describe the surveyed respondents’ characteristics. | 4, Table 1. |
|  |  | • Characterize the questionnaire domains and the number of questions in each domain. | 3 |
|  |  | • Provide details of preserving confidentiality and anonymity | 3 |
|  |  | • Describe pretesting and pilot testing (experts and respondents involved), the number of revision rounds, and the average time for filling out the questionnaire. | 3 |
|  |  | • Report content and face validity (quality, completeness, and feasibility of the questionnaire and its appeal to relevant respondents). | 3 |
|  |  | • Add details of an employed survey platform for web-based surveys (e.g., SurveyMonkey, Google Forms, etc.). | 2, 3 |
|  |  | • Report modes of questionnaire distribution (e.g., via certain social media channels, emails, face-to-face interviews, and postal mail). | 2 |
|  |  | • Clarify when and how many times survey reminders were circulated. | N/A |
| 7 | Adherence to research reporting standards | • Refer to recommendations or their combinations consulted for reporting. | 3 |
| 8 | Ethics section | • Provide ethics committee approval/waiver date, protocol number, and name of the ethics committee. | 2 |
|  |  | • Refer to documents of national health research authorities that regulate the ethics review waiver/exemption. | 2 |
|  |  | • Justify the ethics review exemption in view of the survey's non-interventional origin and absence of informational and psychological risks/harms. | N/A |
|  |  | • Provide details of monetary or other incentives, written informed consents, confidentiality and anonymity, and mechanisms to avoid multiple entries by the same respondents. | 3 |
| 9 | Statistical analyses | • Report descriptive statistics, how categorical data were compared (chi-square or Fisher's exact tests), whether parametric and non-parametric tests and regression analyses were employed, level of significance, and statistical package used. | 4 |
| 10 | Results | • Report response rates in absolute numbers and percentages if the target population was established by methods other than convenience sampling. | 3 |
|  |  | • Reflect on missing data. | 3 |
|  |  | • Provide respondents' details to characterize their representativeness and exclude/minimize nonresponse influence. | 3 |
|  |  | • Insert eye-catching and color graphs and informative tables pointing to the most remarkable results, without recapitulating the same data in the text. | Figure 1 and 2 Table 1 to 5 |
| 11 | Discussion | • Clarify what is new. | 8, 13 |
|  |  | • Analyze limitations by reflecting on low response rate, small sample size, non-response, missing data, a long timeline of collecting responses, language of the questionnaire other than English, and generalizability of the survey results. | 13, 14 |
| 12 | Author contributions and acknowledgements | • Identify the authors who drafted the questionnaire and survey report. | 14 |
|  |  | • List non-author/technical contributions for questionnaire dissemination, promotion, and data collection. | N/A |
| 13 | Disclosure of interests | • Disclose potential conflicts which may affect the validity and reliability of the survey. | 14 |
| 14 | Funding | • Report funding sources, provision of software, and open-access funding, if available. | 14 |
| 15 | Open data sharing | • Add a note about the availability of data for post-publication analyses. | 14 |
| 16 | Appendix | • Submit an English version of the questionnaire. | Supplementary file 1. |

1. Zimba O, Gasparyan AY (2023) Designing, Conducting, and Reporting Survey Studies: A Primer for Researchers. J Korean Med Sci 38:e403. <https://doi.org/10.3346/jkms.2023.38.e403>
